# Identification of potential new treatment response markers and therapeutic targets using a Gaussian process-based method in lapatinib insensitive breast cancer models

**DOI:** 10.1371/journal.pone.0177058

**Published:** 2017-05-08

**Authors:** Tapesh Santra, Sandra Roche, Neil Conlon, Norma O’Donovan, John Crown, Robert O’Connor, Walter Kolch

**Affiliations:** 1 Systems Biology Ireland, University College Dublin, Belfield, Dublin, Ireland; 2 National Institute for Cellular Biotechnology, Dublin City University, Dublin, Ireland; 3 Department of Medical Oncology, St Vincent’s University Hospital, Dublin, Elm Park, Ireland; 4 Conway Institute of Biomolecular and Biomedical Research, University College Dublin, Belfield, Dublin, Ireland; 5 School of Medicine, University College Dublin, Belfield, Dublin, Ireland; University of Nebraska Medical Center, UNITED STATES

## Abstract

Molecularly targeted therapeutics hold promise of revolutionizing treatments of advanced malignancies. However, a large number of patients do not respond to these treatments. Here, we take a systems biology approach to understand the molecular mechanisms that prevent breast cancer (BC) cells from responding to lapatinib, a dual kinase inhibitor that targets human epidermal growth factor receptor 2 (HER2) and epidermal growth factor receptor (EGFR). To this end, we analysed temporal gene expression profiles of four BC cell lines, two of which respond and the remaining two do not respond to lapatinib. For this analysis, we developed a Gaussian process based algorithm which can accurately find differentially expressed genes by analysing time course gene expression profiles at a fraction of the computational cost of other state-of-the-art algorithms. Our analysis identified 519 potential genes which are characteristic of lapatinib non-responsiveness in the tested cell lines. Data from the Genomics of Drug Sensitivity in Cancer (GDSC) database suggested that the basal expressions 120 of the above genes correlate with the response of BC cells to HER2 and/or EGFR targeted therapies. We selected 27 genes from the larger panel of 519 genes for experimental verification and 16 of these were successfully validated. Further bioinformatics analysis identified vitamin D receptor (VDR) as a potential target of interest for lapatinib non-responsive BC cells. Experimentally, calcitriol, a commonly used reagent for VDR targeted therapy, in combination with lapatinib additively inhibited proliferation in two HER2 positive cell lines, lapatinib insensitive MDA-MB-453 and lapatinib resistant HCC 1954-L cells.

## 1 Introduction

BC is the most common type of cancer in women accounting for 25% of all cases [1]. Clinically, BC is classified based on tumour progression, histopathology, and the expression status of HER2, oestrogen, and progesterone receptors. The most suitable treatment for a patient is decided based on these parameters. HER2-positive BC, in which the HER2 receptor is either overexpressed or amplified, accounts for approximately 20–25% of human BC cases [2] and is associated with poor prognosis [3]. Standard treatment options such as radiation, surgery and chemotherapy as well as more targeted approaches are used to treat these types of patients. The monoclonal antibody trastuzumab [4] and the dual tyrosine kinase inhibitor lapatinib are among the targeted therapies currently in clinical use [5]. Here, we focused on lapatinib, which inhibits both HER2 and EGFR and prevents activation of important downstream pathways, such as ERK/MAPK (extracellular-signal-regulated kinase/mitogen-activated protein kinase) and PI3K (phosphatidylinositol 3-kinase) which can drive cancer progression [6, 7]. Lapatinib is currently approved for treatment of metastatic BC in combination with capecitabine [8], and experimentally may synergise with trastuzumab [9]. However, a significant proportion of HER2-positive tumours do not respond to lapatinib. Recent clinical studies suggest lapatinib has a success rate of 12.4–24.7% depending on whether it is administered alone or in combination with capecitabine or trastuzumab [8, 10, 11]. These low response rates underline the clinical urgency to understand and overcome the molecular mechanisms that prevent BC from responding to lapatinib treatment.

Here, we address this question by reanalysing a previously published dataset consisting of gene expression changes in a panel of lapatinib responsive and non-responsive cell lines over a period of time following different doses of lapatinib treatment [12]. We sought to compare the temporal patterns of gene expression between the responsive and non-responsive cell lines in order to identify genes which are characteristic of lapatinib non-responsiveness [12]. Comparing temporal patterns of gene expression across different cell lines/experimental conditions is not straightforward, mainly because of the different types of variabilities in these measurements [13, 14]. Firstly, genotypic differences within different cell populations and measurement errors add random variability to the data [13, 14]. Secondly, gene expression is subjected to various levels of regulation, including epigenetic control, efficiency of transcriptional initiation and extension, and post-transcriptional processing, such as mRNA splicing and degradation. These processes add systematic time dependent variabilities to the measured expression [13, 14]. Standard statistical tools such as t-test, rank sum test etc. which are commonly used to compare gene expression, account for the random variabilities, but fail to capture the systematic temporal variabilities in gene expression data [13, 14]. Several methods have been proposed to model and compare temporal gene expression patterns [15–21]. Gaussian Process (GP) based methods are particularly attractive, since they stem from widely studied, tried and tested theories and are well known to accurately model time course data [16, 17, 19, 21, 22]. In this paper, we developed a fast and accurate GP based differential time course gene expression analysis tool, called GEAGP (Gene Expression Analysis using Gaussian Process), to compare temporal gene expression patterns. We first tested this tool on a simulated dataset and compared its performance with other similar statistical tools. Subsequently, we used this tool to identify potential genes which characterize non-responsiveness of BC cells to lapatinib. Some of the identified genes were then validated by quantitative RT-PCR. Further bioinformatics analysis led to the discovery of a potentially new target for treating lapatinib insensitive cells, the vitamin D receptor (VDR). The potential of the VDR as a new treatment option was then examined in cell line models using concurrent treatment with varying doses of the VDR agonist calcitriol in combination with different doses of lapatinib in lapatinib insensitive and resistant cell lines.

## 2 Materials and methods

### 2.1 Imputing missing values in the microarray data

In the microarray dataset most experiments were performed in quadruplicates, except in a few cases where only three replicates were available. In these cases, we calculated the sample mean (*μ*_*s*_) and standard deviations (*σ*_*s*_) using the three available replicates and then generated a random number by sampling a normal distribution ($N\left(\mu_{s},\sigma_{s}^{2}\right)$) with the same mean and standard deviation. This random number was then used to represent the missing fourth replicate value [23].

### 2.2 Empirical estimation of the GP parameters μ_i_(t), Σ_i_

We assumed that the expression profile of an mRNA is a GP with mean function and covariance matrix *μ*_*i*_(*t*), Σ_*i*_ respectively, i.e. *m*_*i*_(*t*)∼*N*(*μ*_*i*_(*t*), Σ_*i*_). We further assumed that the mean function itself is a GP, i.e. it has the following prior distribution: *μ*_*i*_(*t*)∼*N*(*μ*_0_(*t*), Σ_0_) where *μ*_0_(*t*), Σ_0_ are hyperparameters. Since the distribution of the mean function is unknown a priori, *μ*_0_(*t*) was assumed to be zero, and the Σ_0_ was assumed to be defined by a Ornstein–Uhlenbeck function ($\Sigma_{0}^{t,t^{\prime }}=\psi_{i}\text{exp}\left(-\frac{\left|t-t^{\prime }\right|}{\delta_{i}}\right)$) [24, 25] which is an exponential functional form that describes the correlation between values of *μ*_*i*_(*t*) at different time points (*μ*, *μ*_*i*_). The parameters *δ*_*i*_, *ψ*_*i*_, *d*_*i*_, *v*_*i*_, and $\sigma_{i}^{2}$ of the covariance matrices Σ_0_ and Σ_*i*_ can only take positive values. Therefore, they were assumed to have gamma prior distributions with location and scale parameters *α* = 0.1 and *b* = 100 respectively. The choices of *a*, *b* ensures that the corresponding gamma distributions are flat, thereby allowing a wide range of values for the above parameters to be considered during inference/estimation of *μ*_*i*_(*t*), Σ_*i*_. Multiplying the likelihood (see Eq 2 in the “Results” section) by the prior distributions of *μ*_*i*_(*t*), *δ*_*i*_, *ψ*_*i*_, *d*_*i*_, *v*_*i*_, and $\sigma_{i}^{2}$, and integrating the resulting joint distributions with respect to *μ*_*i*_(*t*) one arrives at the following marginal posterior.

$$p(\delta_{i},\;\psi_{i},\;d_{i},\;v_{i},{\;\sigma }_{\text{i}}^{2}|m_{i}^{r}\left(t\right))=\frac{1}{{\left(2\pi \right)}^{\frac{N_{r}*N_{T}}{2}}\;{\left|\Sigma_{i}\right|}^{\frac{N_{r}}{2}}}\prod_{r=1}^{N_{r}}\text{exp}\left(-\frac{{\bar{\mu_{m}^{r}\left(t\right)}}^{T}{\;\Sigma }_{i}^{-1}\bar{\mu_{m}^{r}\left(t\right)}}{2}\right)\times \Gamma \left(\sigma^{2}|a,b\right)\Gamma \left(\delta_{i}|a,b\right)\Gamma \left(\sigma_{i}|a,b\right)\Gamma \left(d_{i}|a,b\right)\Gamma \left(v_{i}|a,b\right)$$

Where $\bar{\mu_{m}^{r}\left(t\right)}=m_{i}^{r}\left(t\right)-\bar{\mu_{i}\left(t\right)}$ and

$$\bar{\mu_{i}\left(t\right)}={\left(\Sigma_{0}^{-1}+N_{r}\Sigma_{\text{i}}^{-1}\right)}^{-1}\;N_{r}\Sigma_{\text{i}}^{-1}\;m_{a}$$ (1)

We used active set algorithm implemented in MATLAB’s fmincon function (see http://uk.mathworks.com/help/optim/ug/constrained-nonlinear-optimization-algorithms.html for details) to find the values of *δ*_*i*_, *ψ*_*i*_, *d*_*i*_, *v*_*i*_, and $\sigma_{i}^{2}$ which maximizes the above posterior probability. The likelihood of an observed mRNA expression profile was calculated by replacing the maximum a posteriori estimates of *δ*_*i*_, *ψ*_*i*_ in Eq 1, replacing *μ*_*i*_(*t*) in the likelihood function (see Eq 2 in the “Results” section) by $\bar{\mu_{i}\left(t\right)}$ in Eq 1, replacing *d*_*i*_, *v*_*i*_, and $\sigma_{i}^{2}$ in Eq 2 by their maximum a posteriori values.

### 2.3 Simulating time course gene expression profiles

The temporal profiles *m*_*ij*_(*t*) of the *i*^*th*^ gene in the *j*^*th*^ condition were generated by sampling a hierarchical GP with mean and covariance matrix *μ*_*ij*_(*t*), Σ_*ij*_ respectively. The covariance matrix (Σ_*ij*_) has two components $\Sigma_{ij}^{t,t^{\prime }}$, $\Sigma_{ij}^{N}$ to account for systematic and random variabilities, respectively. For simulation, we used a Gaussian kernel function ($\Sigma_{ij}^{t,t^{\prime }}=\text{exp}-\frac{{\left(t-t^{\prime }\right)}^{2}}{l_{ij}}$) to model the systematic variabilities $\left(\Sigma_{i}^{t,t^{\prime }}\right)$ where *l*_*ij*_ is the smoothing parameter. The random noise is Gaussian with *0* mean and variance $\sigma_{ij}^{2}$, i.e. $\Sigma_{ij}^{N}=\sigma_{ij}^{2}I$, *I* is an identity matrix. The smoothing parameter *l*_*ij*_ was sampled from a gamma distribution with shape and scale parameters *a*_*ij*_ and *b*_*ij*_, respectively. The mean function *μ*_*ij*_(*t*) itself was randomly generated by sampling a GP with mean **0** and covariance matrix $\;\Sigma_{ij}^{t,t^{\prime }}$. If a gene (*i*) is not differentially expressed between two conditions (*j* = 1, 2), then *μ*_*i*1_(*t*) = *μ*_*i*2_(*t*) and *a*_*i*1_ = *a*_*i*2_ = 15, *b*_*ij*_ = 1. In the opposite case, i.e. when a gene (*i*) is differentially expressed between two conditions (*j* = 1, 2), we assumed *μ*_*i*1_(*t*) ≠ *μ*_*i*2_(*t*) and these were generated by sampling two corresponding GPs. The smoothing parameters *l*_*i*1_,*l*_*i*2_ were generated by sampling Gamma distributions with the parameters *a*_*i*1_ = 15, *b*_*i*2_ = 1; *a*_*i*2_ = 25, *b*_*i*2_ = 1 respectively.

### 2.4 Gene expression data

The gene expression dataset, which has been described previously, was obtained from GSK in the form of raw data files (.cel files) [12]. Hegde *et al*., treated four cell lines (BT-474, SKBR-3, T47D and MDA-MB-468) with 0.1% DMSO (control), 0.1 μM lapatinib and 1 μM lapatinib at 0, 2, 6, 12, 24 hours. Not all samples that were taken had data for all time-points (see Table 1). All measurements were performed using the U133A Affymetrix human 22,000-element microarray. There were 36 arrays for each cell line, so a total of 144 arrays were used in our analysis. We performed a probe centric analysis, i.e. the intensity values of each Affymetrix probe was modelled using the aforementioned GP model. Once a probe was identified as a potential marker, the corresponding gene was identified using BioMart (http://www.biomart.org/) mapping service.

**Table 1:** Treatment timepoints available from the Hedge *et al*.*’s* dataset.

Cell lines	0.1 μM lapatinib	1 μM lapatinib	0.1% DMSO
**SKBR-3**	6, 12 and 24 hours	6 and 12 hours	0, 2, 6, 12, 24 hours
**BT-474**	2, 6, 12, 24 hours		
**T-47D**		6, 12 and 24 hours	
**MDA-MB-468**			

### 2.5 Cell-lines and reagents

SKBR-3, BT-474, MDA-MB-453, HCC 1954, MDA-MB-468, and T47D were obtained from ATCC and the JIMT-1 cells were obtained from the German Tissue Repository DSMZ. HCC 1954-L was developed in-house through continuous exposure to lapatinib [26]. BC cell lines were maintained in RPMI 1640 medium supplemented with 10% fetal bovine serum (Gibco). HCC 1954-L were maintained in culture in 1μM lapatinib, however, cells were removed from drug 7 days prior to all assays. All cell lines were maintained at 37°C in a 5% CO_2_ incubator.

Calcitriol and lapatinib were obtained from Sequoia Research Products (Pangbourne UK). Cell culture media, DMSO, ethanol were obtained from Sigma-Aldrich (Dublin, Ireland).

Lapatinib stock solution (10 mM) was prepared in DMSO. Calcitriol stock solution (10 mM) was prepared in ethanol and stored at -20°C.

### 2.6 Lapatinib treatment and RNA extraction

Triplicate RNA samples were prepared from SKBR-3, BT-474, MDA-MB-468, T47D, MDA-MB-453 and JIMT-1. The cell lines were grown to approximately 75% confluency. Test samples were treated with 1 μM lapatinib for 16 hours. Control samples were treated with equivalent concentrations of DMSO. After the 16 hour incubation, RNA was extracted from the control and treated samples using the RNeasy mini Kit (Qiagen, Hilden, Germany) according to the manufacturer’s protocol and were treated with RNase-free DNase (Qiagen).

RNA was isolated from MDA-MB-453 and JIMT-1 cells using TriReagent (Sigma, Ireland). The RNA was extracted using a liquid-liquid extraction procedure with chloroform (0.2 mL) and iso-propanol. The RNA was washed with 75% ethanol and resuspended in 40 μL RNase free water.

cDNA was prepared from 2 μg of total RNA using a high capacity RNA to cDNA kit (Applied Biosystems, Foster City, CA, USA).

### 2.7 Taqman RT PCR

Taqman Array 96 well fast plates (Applied Biosystems, Dublin Ireland) were pre-coated with primers for the specific genes for validation *(ALDH3A2*, *AMD1*, *ANKRD10*, *ATP2B4*, *COPS3*, *EIF2S2*, *EIF4B*, *GAPDH*, *GTPBP4*, *GUSB*, *IGBP1*, *ING4*, *KLHDC2*, *KLHL24*, *MPZL1*, *NUDC*, *PPIC*, *PPP2CA*, *PTP4A1*, *PTP4A2*, *RB1CC1*, *RNF13*, *SOCS3*, *SPAG1*, *TFAP2C*, *TIMM17A*, *TJP2*, *VDR*, *WIPI2*, *ZDHHC17)*.

40 ng of cDNA and 5 μL of Taqman Fast Universal Master Mix (2x), no AmpErase UNG (Applied Biosystems) were dispensed into each well.

The following thermal cycling specifications were performed on the ABI 7900 Fast Real-Time PCR system (Applied Biosystems, Foster City, CA, USA); 20 s at 95°C and 40 cycles each for 3 s at 95°C and 30 s at 60°C. Expression values were calculated using the comparative cycle threshold (Ct) method [27]. 18S ribosomal RNA was selected as the endogenous control. The cycle threshold (Ct) indicates the cycle number by which the amount of amplified target reaches a fixed threshold. The Ct data for 18S was used to create ΔCt values [ΔCt = Ct (target gene)-Ct (18S)]. ΔΔCt values were calculated by subtracting ΔCt of the calibrator (DMSO controls) from the ΔCt value of each target. Relative quantification (RQ) values were calculated using the equation 2-ΔΔCt. Differences in the mRNA expression level between untreated and drug treated samples were assessed using the Students t test.

A VDR gene expression assays (Hs01045840_m1) was performed with MDA-MB-453 and JIMT-1 cDNA (5 ng/well), using 18S as (Hs99999901_S2) as endogenous control.

### 2.8 Proliferation assay in vitro

Proliferation was measured using an acid phosphatase assay [28]. 2.5 to 5 x 10^3^ cells per well were seeded in 96 plates and incubated for 24 hours prior to the addition of drug. After 5 days of drug treatment cells were washed with PBS. 10 mM paranitrophenol phosphate substrate (Sigma-Aldrich) in 0.1 M sodium acetate buffer with 0.1% Triton X (Sigma Aldrich) was added to each well and incubated at 37°C for 2 hours. The reaction as stopped with 50 μL of 1 M NaOH and the absorbance was read at 405 nM (reference—620 nM). Growth of drug treated cells was calculated relative to control untreated cells in biological triplicate.

## 3 Results

### 3.1 Comparing time course gene expression profiles using GP

Time course gene expression data consists of noisy measurements of mRNA levels at different time points. As discussed before, these measurements contain both systematic and random variabilities. It is necessary to take both types of variabilities into account when comparing two sets of time course measurements of mRNA expression. We developed an empirical GP model of time course mRNA profiles that captures (a) average mRNA levels at different points in time, (b) the extent of random uncertainty caused by cell to cell variability and experimental measurement noise; and (c) the temporal variability in mRNA profiles caused by internal mechanisms of gene regulation. In our formulation, a time course mRNA profile (*m*_*i*_(*t*)) is assumed to have a Gaussian distribution with a mean (*μ*_*i*_(*t*)) which represents the average mRNA levels at different time points (*t*), and a covariance matrix (Σ_*i*_) which captures the overall variability in the mRNA profile. The covariance matrix (Σ_*i*_) has two components ($\Sigma_{i}=\Sigma_{i}^{t,t^{\prime }}+\Sigma_{i}^{N}$), one ($\Sigma_{i}^{t,t^{\prime }}$) accounts for systematic variability between mRNA expressions at any two instants of time (*t*, *t'*) and the other ($\Sigma_{i}^{N}$) accounts for random noise. We used a modified Ornstein–Uhlenbeck function ($\Sigma_{i}^{t,t^{\prime }}=v_{i}\text{exp}\left(-\frac{\left|t-t^{\prime }\right|}{d_{i}}\right)$) [24, 25, 29], to model the systematic variabilities $\left(\Sigma_{i}^{t,t^{\prime }}\right)$ in mRNA expressions between two instants in time (*t*, *t'*), where *v*_*i*_ and *d*_*i*_ are two parameters [30]. The random noise is assumed to be Gaussian with *0* mean and variance $\sigma_{i}^{2}$ (i.e. $\Sigma_{i}^{N}=\sigma_{i}^{2}I$, *I* is an identity matrix). Under these assumptions, the likelihood of a set of observed mRNA profiles ($m_{i}^{r}\left(t\right)$) is given by

$$p\left(m_{i}^{r}\left(t\right)|\mu_{i}\left(t\right),\Sigma_{i}\right)=\frac{1}{{\left(2\pi \right)}^{\frac{N_{r}*N_{T}}{2}}\;{\left|\Sigma_{i}\right|}^{\frac{N_{r}}{2}}}\prod_{r=1}^{N_{r}}\text{exp}\left(-\frac{\mu_{m}^{r}{\left(t\right)}^{T}\Sigma_{i}^{-1}\mu_{m}^{r}\left(t\right)}{2}\right)$$ (2)

where *N*_*r*_ is the number of replicates and $\mu_{m}^{r}\left(t\right)=m_{i}^{r}\left(t\right)-\mu_{i}\left(t\right)$. *μ*_*i*_(*t*) and Σ_*i*_ are estimated from observed data as shown in Materials and Methods (M&M).

We designed a statistical hypothesis test to see whether two sets of ($m_{i}^{c_{1}}\left(t\right),m_{i}^{c_{2}}\left(t\right)$) expression profiles of an mRNA, measured under two experimental conditions (*c*_1_, *c*_2_), are significantly different from each other. In this test we compared the likelihoods (Eq 2) of two hypotheses: (a) both sets of mRNA profiles ($m_{i}^{c_{1}}\left(t\right),m_{i}^{c_{2}}\left(t\right)$) have the same GP model ($m_{i}^{c_{1}}\left(t\right),m_{i}^{c_{2}}\left(t\right)\sim GP\left(\mu_{i}\left(t\right),\Sigma_{i}\right)$); and (b) they come from two different sets of GP models ($m_{i}^{c_{1}}\left(t\right)\sim GP\left(\mu_{i}^{c_{1}}\left(t\right),\Sigma_{i}^{c_{1}}\right),m_{i}^{c_{2}}\left(t\right)\sim GP\left(\mu_{i}^{c_{2}}\left(t\right),\Sigma_{i}^{c_{2}}\right)$)). The comparisons were performed using a likelihood ratio test. The test was repeated for each mRNA in the dataset, producing a set of p-values. Subsequently, the Benjamini-Hochberg method was used to correct for multiple testing, and differentially expressed mRNAs were selected within a 5% false discovery rate (FDR). These mRNAs are the ones whose temporal expression patterns are significantly different between the two experimental conditions under investigation. An implementation of the above pipeline, named GEAGP, in MATLAB is freely available for download from https://github.com/SBIUCD/GEAGP.git.

### 3.2 Comparing the performance of GEAGP with other methods

To evaluate and compare the performance of the GEAGP framework with other methods we generated 100 simulated datasets. Each simulated dataset consists of the expression profiles of 1000 genes measured at 10 equal intervals over a period of 100 hours in two different conditions. Four replicates were generated for each expression profile. Approximately half of the genes in each dataset were randomly chosen to have differential expression patterns between the two conditions. For the simulation, we assumed that the genes which are not differentially expressed have the same underlying pattern (average expression at each time point) with the same level of added systematic and random variabilities in each replicate across the two conditions. On the other hand, the genes which are differentially expressed were assumed to have different underlying patterns with different levels of systematic variabilities across the two conditions. The random variability, which represents biological variability and measurement errors, was kept at the same level in both conditions. Details of the data simulation process are described in the methods section. Random noise was kept at a moderate level (standard deviation = 0.2).

Then, we used four different methods to find differentially expressed genes in each simulated dataset. Two of these methods, ttest & ranksum test, are widely used to analyse static gene expression data. The other two methods, GEAGP and GPREGE [17], use GP to analyse temporal expression profiles. The main differences between GEAGP and GPREGE [17] are the following: GEAGP directly estimates the mean functions of the GP from data, whereas GPREGE assumes that the mean function is a linear combination of a set of basis functions, and attempts to infer the coefficients of these linear equations. The two methods also use different mathematical formulations for parameter estimation and optimization process (see M&M and [17] for details). Prediction accuracy of each method was evaluated by calculating the area under the receiver operating characteristic (AUROC) curve (for details see [31]) based on the results produced by these methods on each dataset. AUROCs can have values between 0 and 1, and the closer these values are to 1 the better is the accuracy of the inferred networks, with AUROC = 1 being the ideal case. The average AUROCs across all 100 datasets represents the overall prediction accuracy of a method and the corresponding standard deviation represents the confidence intervals surrounding these estimates. The results of the above analysis suggest that GEAGP and GPREGE have comparable accuracies (AUROCs = 0.946 ± 0.0074 and 0.952 ± 0.0046 respectively), whereas both t-test and ranksum test were less accurate than the GP based methods (average AUROCs = 0.7538± 0.0087 and 0.7432 ± 0.0082 respectively). Although the accuracies of GPREGE and GEAGP are comparable, GEAGP has the distinct advantage of speed. In the above dataset GEAGP and GPREGE took on an average ≈ 0.5 and ≈ 7.5 seconds, respectively, to execute each differential expression test on a Intel Core i7 (3rd Gen) 3720QM based laptop with 20Gb RAM, i.e. GEAGP is on an average about 15 times faster than the GPREGE. To illustrate what it means for a medium scale bioinformatic analysis, consider a dataset consisting 20,000 genes and 10 experimental conditions. To find differentially expressed genes between each pair of conditions one needs to perform $\left(\begin{array}{c} 10\\ 2 \end{array}\right)=45$ differential expression tests for each gene. Using a single core of the Intel Core i7 (3rd Gen) 3720QM processor, GEAGP will take approximately 5 days to do this analysis, whereas GPREGE will take more than 78 days. Using all four cores of the processor GEAGP will finish the task in about 1 days and 8 hours, whereas GPREGE will take more than 19 days. Thus, GEAGP provides similar accuracy as GPREGE at a fraction of the computational cost, making it more suitable for the analysis of medium to large scale time course data sets. Encouraged by the above results, we used GEAGP to analyse real gene expression datasets.

### 3.3 Discovering markers of lapatinib insensitivity in BC cells using GEAGP

GEAGP was used to analyse time course mRNA profiles in BC cell lines which were treated with different doses of lapatinib and DMSO [12]. In this experiment the mRNA expression profiles were measured in two lapatinib responsive (BT-474, SKBR-3) and two lapatinib non-responsive (MDA-MB-468, T47D) BC cell lines. HER2 and EGFR, the two receptor kinases targeted by lapatinib, have different expression patterns in these cell lines (Table 2).

**Table 2:** Histological-subtypes, HER2 and EGFR receptor expressions and mutation status of SKBR-3, BT-474, T47D and MDA-MB-468 cell lines.

		Protein Expression		Gene Mutation Status						
Cell line	Subtype	EGFR	HER2	*KRAS*	*BRAF*	*PI3K*	*RB1*	*p53*	*PTEN*	*ERBB2*
SKBR-3	HER2 +ve	-	-					R175H		
BT-474	HER2 +ve	38.65	97.99			K111N		E285K		AMPL
T47D	Luminal A	44.35	77.55			H1047R		L194F		
MDA-MB-468	Basal Like	99.66	39.05				DEL	R273H	E545A	

The percentile scores indicate the percentage of cell lines in GDSC database which had similar or lower expressions of the same genes. The first column contains cell-line name. Second column contains histological subtypes, third and fourth columns contain expression of EGFR and HER2 receptors. The expressions are given in percentiles, i.e. percent of cell-lines in the GDSC database which have similar or lower expressions of HER2 or EGFR than the cell line corresponding to the percentile value. Fifth till eleventh columns contain mutation status. Empty cells represent no mutation, DEL represents deletion, AMPL represents amplification, and the remaining non-empty cells contains amino acid substitution information.

HER2 is over-expressed in both lapatinib responsive cell lines SKBR-3 and BT-474 [32]. EGFR is overexpressed in SKBR3 [33], but has relatively low expression in BT-474 (~62% of 1018 cancer cell lines in the GDSC database had higher EGFR expression than BT-474) [34]. The non-responsive cell lines, T47D and MDA-MB-468 are classified as luminal A and basal-like BC cells in literature [32]. Both types of BC cells have low HER2 expression. However, T47D has high HER2 expression (more than 77.55% of GDSC cell lines [34]) and moderate low EGFR expression (lower than ~56% of GDSC cell lines [34]). MDA-MB-468 cells have low HER2 expression (lower than ~61% of GDSC cell lines [34])) and very high level of EGFR expression (higher than ~99.6% of all GDSC cell lines [34]). We also looked at the mutation spectrum of these cell lines using the COSMIC database (cancer.sanger.ac.uk). Data on some of the mutations which were previously implicated in lapatinib resistance are shown in Table 2. Among the responsive cell lines, SKBR3 has a p53 mutation, whereas, BT-474 has PI3K, ERBB2 and p53 mutations. Among the non-responsive cell lines, T47D has a p53 and PI3KCA mutation, whereas MDA-MB-468 has p53, PTEN and RB1 mutations. None of the cell lines have KRAS or BRAF mutation. Therefore, there is no particular set of mutations that separate the above responsive and non-responsive cell lines. This suggest that the expressions of EGFR and HER2 receptors and/or the mutation statuses do not explain the responsiveness of the above cell lines to lapatinib treatment. Hence, these cell lines are reasonable choices for studying lapatinib response in BC cells.

The cells were treated with 0.1 μM DMSO, 0.1 μM or 1 μM lapatinib[12]. The measurements were taken at 0, 2, 6, 12 and 24 hours for DMSO treated cells; at 2, 6, 12, 24 hours for cells treated with low doses (0.1μM) of lapatinib (except for SKBR-3 cells for which no measurements were available at 2 hours); and at 2, 6 and 12 hours for cells treated with high doses (1.0 μM) of lapatinib (except for BT-474 and SKBR-3 cell lines for which measurements were not available at 2 hours). Each experiment was replicated 3–4 times. Missing replicates (where only 3 replicates were available instead of 4) were imputed based on the intensities observed in the other replicate experiments (see M&M for details). The resulting dataset was then used to identify potential markers of lapatinib insensitivity in BC cells.

We assumed that genes which satisfy all of the following criteria are potential markers for lapatinib insensitivity:

- Have similar expression profiles within the responsive or non-responsive groups of cell lines, but are differentially expressed between these two groups.
- Have altered expression in response to both doses of lapatinib treatment in responsive cells but not in non-responsive cells.

**Fig 1:**
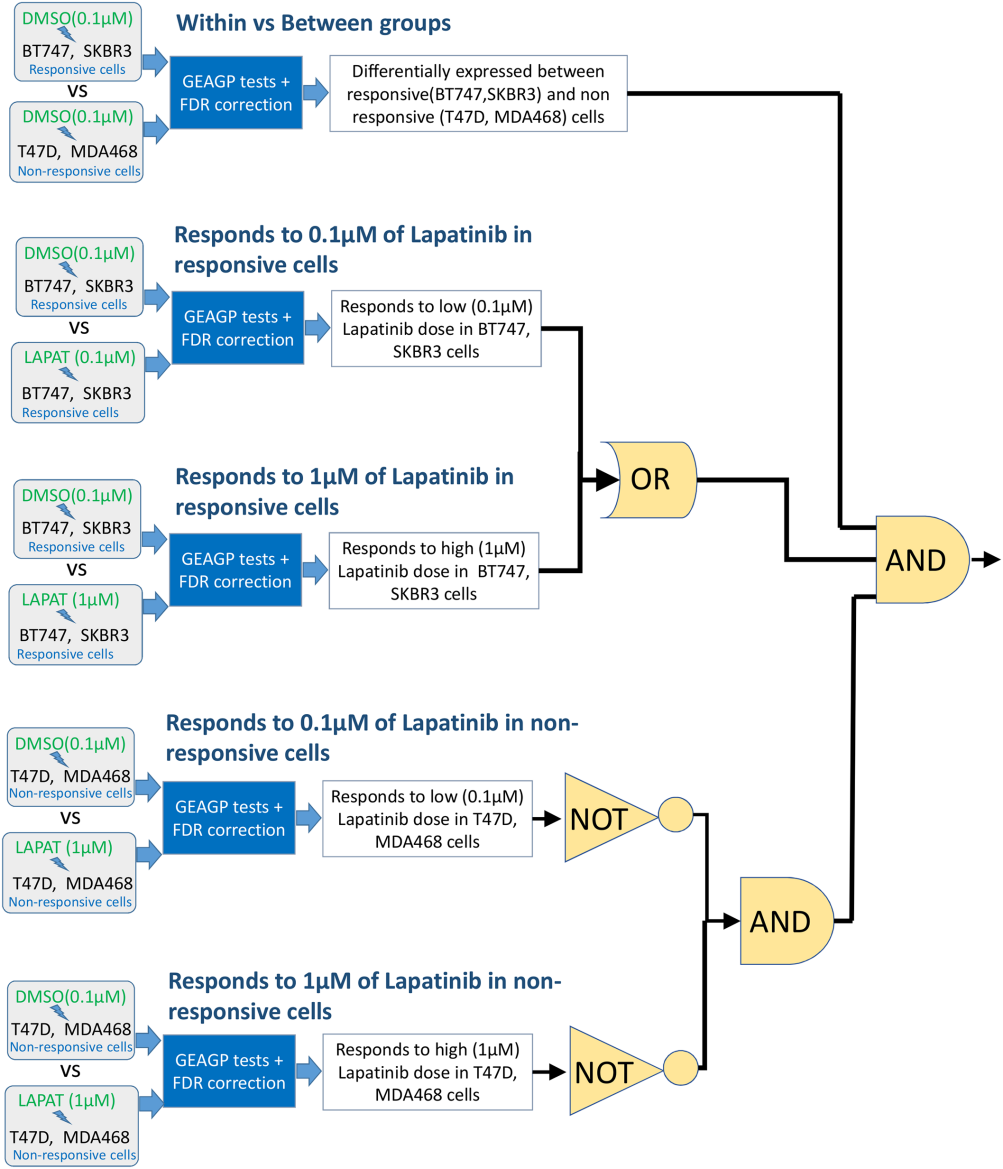
A schematic diagram of the GP based analysis performed on the gene expression dataset of [12].

To find genes which satisfy the above criteria we performed a two stage analysis. In the first stage each mRNA expression profile was subjected to the following hypothesis tests (Fig 1):

- **Responsive vs non-responsive groups (Test 1):** The objective of this test was to identify genes which have differential expression patterns between DMSO treated responsive (SKBR-3, BT-474) and non-responsive cell lines (MDA468, T47D). Here we tested the hypothesis that a single GP model is sufficient to represent the mRNA expression profiles of a gene in all four cell lines when the cells are treated with DMSO, vs the alternative hypothesis that two GP models are needed, one for the responsive cell lines and another for the non-responsive cell lines.
- **Responds to 0.1μM of lapatinib in responsive cells (Test 2):** This test aimed to identify genes which have differential expression patterns between DMSO treated and 0.1 μM lapatinib treated responsive cells (BT-474, SKBR-3). Here we tested the hypothesis that one GP model is sufficient to model the mRNA expression profiles of a gene in both DMSO and lapatinib (0.1 μM) treated responsive cell-lines (BT-474, SKBR-3) against the alternative hypothesis that two separate GP models are needed, one for DMSO treated cells and the other for lapatinib (0.1 μM) treated cells.
- **Responds to 1 μM of lapatinib in responsive cells (Test 3):** The goal of this test was to identify genes which have differential expression patterns between DMSO and 1 μM lapatinib treated responsive cells (BT-474, SKBR-3). Here, we tested the hypothesis that the one GP model is sufficient to model the mRNA expression profiles in DMSO and lapatinib (1 μM) treated responsive cells (BT-474, SKBR-3) against the alternative hypothesis that two separate GP models are needed, one for DMSO treated cells and the other for lapatinib (0.1 μM) treated cells.
- **Responds to 0.1 μM of lapatinib in non-responsive cells (Test 4):** This test identified genes whose expression is altered in response to low doses (0.1 μM) of lapatinib in non-responsive cells (MDA468 and T47D). Here, we tested the hypothesis that one GP model is sufficient to model the mRNA expression profiles in DMSO and lapatinib (0.1 μM) treated non-responsive cells (MDA468 and T47D) against the hypothesis that two separate GP models are needed, one for DMSO treated cells and the other for lapatinib (0.1 μM) treated cells.
- **Responds to 1μM of lapatinib in non-responsive cells (Test 5):** This test identified the genes whose expression in altered in response to high doses (1 μM) of lapatinib in non-responsive cells (MDA468 and T47D). This tested the hypothesis that only one GP model is sufficient to model the mRNA expression profiles in DMSO and lapatinib (1 μM) treated non-responsive cells (MDA468 and T47D) against the hypothesis that two separate GP models are need, one for DMSO treated cells and the other for lapatinib (0.1 μM) treated cells.

The genes which passed the first test and at least one of the second and third tests, but fail both of the last two tests within a 5% false discovery rate were assumed to satisfy the criteria of genes selectively regulated by lapatinib in responsive, but not in non-esponsive cells. This analysis selected 519 genes out of 22000 genes as potential markers of lapatinib response (Table A in S1 File). In a second step, these genes were further analysed using established bioinformatics methods as described below.

### 3.4 Bioinformatics analysis of markers of lapatinib insensitivity in BC cells

**Fig 2:**
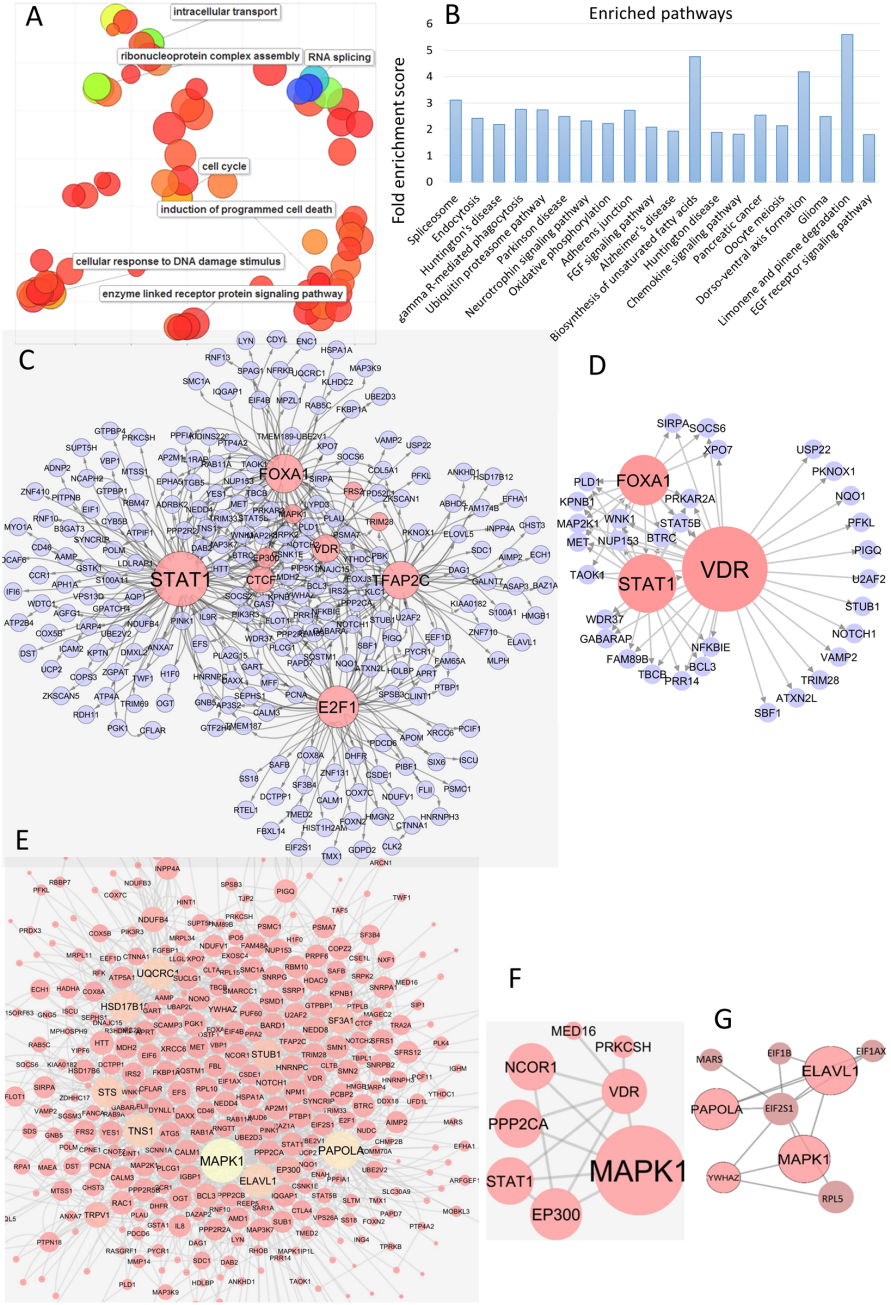
Bioinformatics analysis of the selected genes. (A) Enriched gene ontology terms are summarized and visualized by REVIGO. Here, each circle represents a gene ontology term and the size of the circle represents the extent of enrichment. (B) Pathway enrichment analysis of the selected genes. Pathways are shown in X-axis and the enrichment scores are shown in Y-axis. (C) Transcriptional module found in the identified genes. (D) Transcriptional activity of VDR. (E) PPI network induced by the identified genes. (F,G) PPIs of VDR and EIF2S2.

First, we performed gene ontology enrichment analysis on the 519 selected genes using DAVID [35, 36]. The enriched gene ontology terms were then summarized and visualized by REVIGO (Fig 2A) [37]. The most enriched gene ontology terms fall into seven main categories, i.e. (i) cell cycle, (ii) apoptosis or programmed cell death, (iii) DNA damage, (iv) RNA splicing, (v) intracellular transport, (vi) signal transduction and (vii) chromosome assembly. These biological processes are usually found altered in cancer cells, and therefore it is not surprising to see genes which participate in these process responding differentially to lapatinib treatment in responsive and non-responsive BC cells. We also performed pathway enrichment analysis using DAVID [35, 36] and employing KEGG [38] and PANTHER pathway databases [39]. Several signalling pathways that play key roles in cancer, e.g. EGFR pathway, FGF pathway, spliceosome, proteasome, FcγR mediated signalling, were also found to be enriched in the selected genes (Fig 2B). FGF and FcγR share several components with EGFR pathway which is activated by the EGFR family of receptors, two of which are targets of lapatinib. Ten genes, RAC1, STAT1, YWHAZ, PPP2CB, MAP2K1, MAPK1, PPP2R5B, PLCG1, PPP2CA, STAT5B, belonging to the EGFR pathway were in the list of potential markers of lapatinib insensitivity.

We then looked for known transcription regulatory interactions (TRIs) among the selected genes. Human genome wide known TRIs were retrieved from two sources, the ENCODE database containing ChIP (chromatin immunoprecipitation) on ChIP data [40–42] and the HTRIB database [43] which contains experimentally validated TRIs. All TRIs, where both the transcription regulator and its target gene were identified as potential markers for lapatinib insensitivity in the aforementioned GEAGP analysis, were retrieved. The ensemble of these TRIs (Fig 2C) represents part of the known human transcription regulatory network which is dysregulated in lapatinib non-responsive BC cells. Interestingly, this dysregulated network consists of a transcriptional module formed by eight transcription factors STAT1, CTCF, EP300, FOXA1, VDR, MAPK1, TFAP2C, E2F1 (Fig 2C) which regulate each other and a large number of the genes selected by the GEAGP analysis. Among these transcription factors, STAT1, MAPK1, FOXA1, TFAP2C, E2F1 are associated with resistance or insensitivity to several anticancer therapies including tyrosine kinase inhibitors such as lapatinib in BC cells [44–51]. CTCF and EP300 also have been linked to BC. The putative tumour suppressor gene EP300 is frequently mutated in BC [52, 53], while CTCF overexpression is correlated with resistance to apoptosis in BC [52, 53]. Polymorphism of the VDR (Vitamin D Receptor) was previously associated with BC [54], but its expression has no known association either with BC or with lapatinib insensitivity. Since it was found to be part of the same transcriptional module whose constituents are associated with lapatinib insensitivity, VDR could be a potential target for modulating lapatinib response in BC cells which do not respond to lapatinib.

We further investigated the protein-protein interaction (PPI) network formed by the protein products of the selected genes. The STRING database [55] was used to find PPIs between the products of the selected genes (Fig 2E). Interestingly, many of the above transcription factors (MAPK1, TFAP2C, EP300) act as large hubs in the PPI network. They were also found to interact with each other, not only at the transcriptional level as found in the TRI network, but also at the protein level (Fig 2E–2G). In the PPI networks, VDR interacts with relatively few proteins (Fig 2F). However, its interactors include STAT1, MAPK1 and PPP2CA, all of which have been associated with lapatinib response [44–46, 56]. This strengthens the case for VDR as a potential target for influencing lapatinib insensitivity. Another interesting protein is EIF2S2. It interacts with several large hubs such as MAP2K1, ELAVL1 and PAPOLA1 in the PPI network (Fig 2G), and therefore may play important roles in insensitivity of BC cells to cancer drugs.

The above analysis suggests that many of the genes which were identified by the GEAGP algorithm have association with BC and lapatinib insensitivity. Additionally, it also demonstrates that some of these genes play important role in the transcriptional and protein interaction networks that are dysregulated in lapatinib insensitive BC cells, and therefore can be potential targets for treating these cells.

### 3.5 Validating the markers of lapatinib insensitivity using GDSC data

We analysed the GDSC database [34] to see if the expression of the genes selected by the GEAGP algorithm correlates with responses of BC cells to HER2 and/or EGFR targeted therapies. Any such correlation will strengthen their potential as markers for BC cell response to HER2 and/or EGFR targeted therapies. GDSC database [34] contains basal gene expression and drug response data for fifty BC cell lines belonging to different histological subtypes and five drugs (lapatinib, erlotinib, EKB-569, afatinib and CP724714) that target HER2 and/or EGFR tyrosine kinases. Expression data was available for 458 of 519 genes selected by the GEAGP algorithm. We calculated pearson correlation between the mRNA expressions of each gene and the area under the dose response curves of each cell-line for each drug. mRNA expressions of 120 genes out of 458 had statistically significant (p-value<0.05) correlation with the responses of BC cells to at least one of the five drugs (Table B in S1 File). When corrected for false discovery rate (FDR), 32 of the 120 genes were selected at 10% FDR (Table B in S1 File). Most of these genes’ expressions correlated with response to afatinib which, like lapatinib, targets HER2 and EGFR. There were relatively little correlation between the expressions of these genes and lapatinib response in GDSC dataset [34]. This is most likely due to lack of lapatinib response data which is available for only 13 out of 50 BC cell lines. Nevertheless, the above analysis suggests that many of the genes that were identified as markers of lapatinib insensitivity in T47D and MDA-MB-468 cells, can indeed be generally used as markers of BC cell response to a variety of HER2 and/or EGFR targeted therapies.

### 3.6 Experimental validation of selected genes

It is not feasible to experimentally validate all 519 genes which were identified in the above analysis due to time and cost factors. To select a smaller subset for experimental validation, we first compared our list of potential lapatinib insensitivity markers to that determined in a previous study by O'Neill *et al*.[57]. O’Neill *et al*. used multiple parallel t-tests to analyse part of the same microarray dataset used here for identifying potential lapatinib biomarkers. 25 genes were found to be common between our list and that of the O’Neill *et al*.‘s study. These common genes include

- VDR, EIF2S2 and SOCS3 (regulator of STAT1) which were found to play important roles in the PPI and transcriptional networks in the bioinformatics analysis
- ANKRD10, PTP4A1, PTP4A2, VDR, ATP2B4 which had statistically significant correlation with BC cell responses to HER2 and/or EGFR targeted therapies

Therefore, this list includes a mix of genes identified in the bioinformatics analysis, GDSC data [34] analysis, and a set of new lapatinib insensitivity markers. We further added two more genes to this list, phosphatase PPP2CA and transcription factor TFAP2C, both of which was identified to be important in the bioinformatics analysis and the later also has statistically significant correlation with BC cell responses to HER2 and/or EGFR targeted therapies (Table B in S1 File).

**Fig 3:**
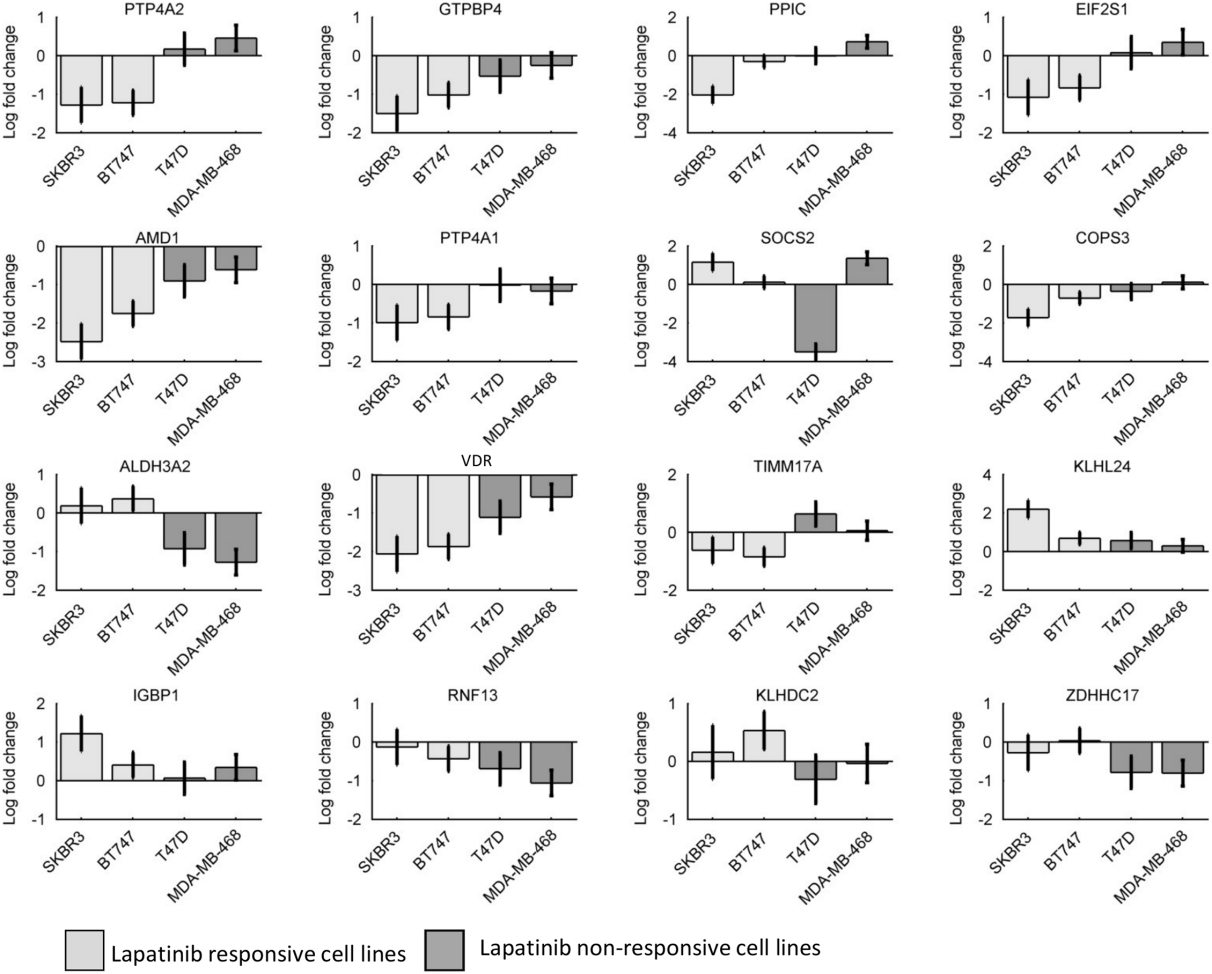
Real time qPCR based validation of a set of selected potential biomarkers. Each panel consists of the log fold changes (lapatinib treated vs untreated, shown in Y-axis) of the corresponding gene (shown in the header of each panel) in all four cell lines (X-axis). Plots are based on N = 3 biological replicates.

The differential gene expression levels of the resulting 27 genes (see Table 1) and an endogenous control gene (18S ribosomal RNA) were measured in mRNA from SKBR-3, BT-474, T47D and MDA-MB-468 cell lines treated with DMSO or lapatinib (1 μM) for 16 hours, using real time quantitative polymerase chain reaction (qRT-PCR). The data obtained from the above experiment were then statistically analysed to examine whether expression of these genes was differentially altered in response to lapatinib in responsive and non-responsive cell lines. Relative expression values were calculated using the comparative threshold cycle (*C*_*T*_) method [27]. First, we subtracted the cycle threshold (*C*_*T*_) values of the housekeeping gene (18S) from those of the selected genes [58]. The resulting values (Δ*C*_*T*_) represent the responses of each gene to DMSO/ lapatinib and were analysed using ANCOVA (Analysis of Covariance) analysis [59]. For the ANCOVA analysis, the Δ*C*_*T*_ values of each gene were used as response variables, the dose of lapatinib was used as predictor variable (1 μM lapatinib for treated cell, 0 μM for DMSO treated control cells), and the responsive or non-responsive (to lapatinib) status of the cell lines were used as covariates. The p-values calculated by the ANCOVA analysis were then subjected to correction for multiple testing. For this purpose, a false discovery rate (FDR) corresponding to each p-value was calculated using the Benjamini Hochberg method. Genes with FDR lower than 0.1 (representing 10% false discovery rate) were considered to pass the validation test. We used MATLAB functions aoctools and mafdr for the ANCOVA and FDR analysis, respectively. 16 out of 28 genes passed the test and their changes (in terms of average log-fold change with respect to DMSO treated cells) to lapatinib treatment in all four cells lines are shown in Fig 3.

### 3.7 Identifying potential therapeutic targets for treating lapatinib insensitive BC cells

To find potential targets, we focussed on the genes which satisfy the following criteria:

- identified by GEAGP as potential lapatinib insensitivity markers
- have statistically significant correlation (at <10% FDR) with BC cell response to HER2 and/or EGFR targeted therapy.

32 genes (Table B in S1 File) satisfy these conditions. To further narrow down the list of potential targets we investigated whether the expressions of any of these genes correlate with BC patient survival. Ideally, we would like to analyse data from BC patients who was treated with HER2 and/or EGFR targeted therapies. But, due to lack of sufficient details on treatment history of patients, we analysed data from HER2 negative, and basal type BC patients who typically do not respond to lapatinib therapies [60, 61]. In some cases, HER2 positive patients do not respond to these types of therapies due to lack of EGFR expression or acquired resistance [62]. Therefore, we also included HER2 positive patients in our survival data analysis. We used Kaplan-Meier plot to find association between expression of each gene in the list (Table B in S1 File) and recurrence free survivals of HER2 negative, basal-like and HER2 positive patient cohorts. We used kmplot [63] web tool which integrate data from several public sources to perform this analysis. 26, 21 and 23 genes (out of total 32) were associated at 10% FDR with the survival of HER2 negative, basal and HER2 positive patient cohorts (Table C in S1 File). 14 genes, SDS, UCP2, KPNB1, REEP5, ALAS3, ZNF10, SDC1, GPAA1, RHOB, EXOSC4, FOXA1, VDR, GLANT7, MLPH, had significant association with the survival of all three patient cohorts. We did not find any reference to REEP5, ALAS3, ZNF10, EXOSC4 and GLANT7 in existing BC literature. Therefore association of these genes to BC, especially lapatinib insensitivity of BC cells, may be novel finds of this study. SDS, UCP2, KPNB1, SDC1, GPAA1, and MLPH had been sporadically studied in the context of BC [64–69]. But, to the best of our knowledge, their role in lapatinib insensitivity of BC cells are not known. VDR, FOXA1 and RHOB are well studied in the context of BC. However, to the best of our knowledge, their potential as a therapeutic target for treating lapatinib insensitive BC cells was not previously investigated. We selected VDR for further experimental testing.

### 3.8 Investigation of VDR as a therapeutic target

**Fig 4:**
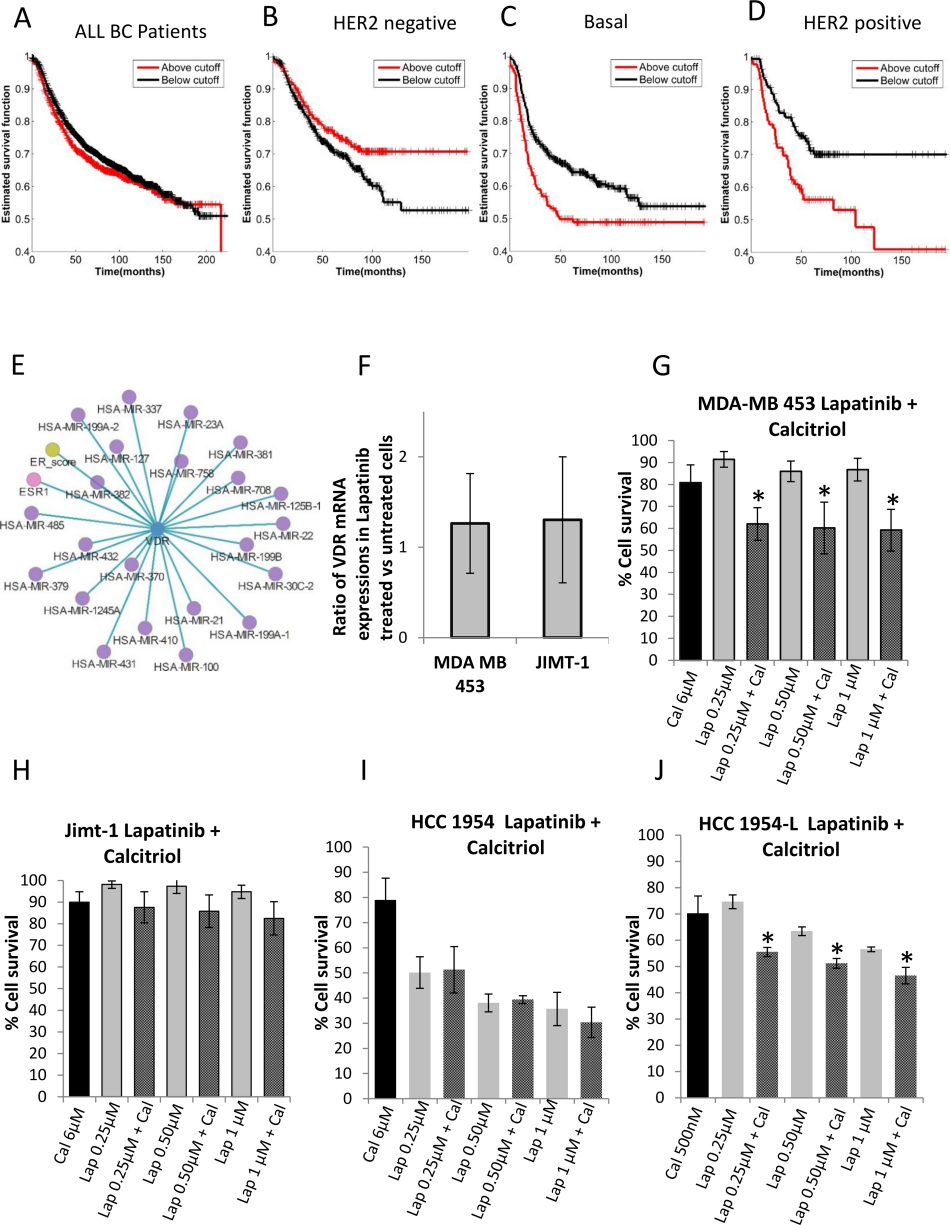
Interrogation of VDR as potential therapeutic targets for sensitizing lapatinib insensitive breast cancer cell lines to lapatinib treatment. (A-D) Association between recursion free survival of all, HER2 negative, triple negative, HER2 positive breast cancer patients with VDR expression. (E) Association between VDR expression and the expressions of 22 miRNAs in the TCGA breast cancer patient cohort. (F) ΔΔ*C*_*T*_ values for Vitamin D Receptor in HER2 positive lapatinib insensitive cell lines (MDA-MB-453 and JIMT-1) showing changes between untreated cells and cells expose to lapatinib for 16 hours. (G-J) Combined treatment with lapatinib and calcitriol in MDA-MB-453, JIMT-1, HCC 1954 and HCC 1956-L cells respectively (* denotes p<0.05).Plots are based on N = 3 biological replicates.

Vitamin D Receptor (VDR), a member of the nuclear receptor family of proteins, acts as a receptor for active vitamin D as well as a DNA-binding transcription factor. In that respect, it is similar to more familiar cancer targets such as oestrogen receptor, androgen receptor and progesterone receptor [70]. Patient survival analysis using data from kmplot database [63] suggests that VDR expression does not correlate with recurrence free survival of BC patients in general (Fig 4A). However, HER2 negative BC patients (typically do not respond to lapatinib) with high VDR expressions are more likely to survive longer than those with low VDR expressions (Fig 4B). Interestingly, basal-like BC patients, most of whom have low or no HER2 expression show the opposite trend, that is, patients with low VDR expression are more likely to survive longer than patients with high VDR expression (Fig 4C). HER2 positive patients exhibit similar trend as the basal-like patients (Fig 4D). Furthermore, using Regulome explorer (http://explorer.cancerregulome.org/) we investigated whether VDR expression has any significant association with other genomic features of BC patients. It was found to have significant correlations with protein expression of Estrogen receptor and the expressions of twenty two miRNAs (Fig 4E), most of which had been previously implicated in proliferation, apoptosis, senescence, resistance to drugs including HER2 targeted therapies [71, 72]. This further strengthens VDR’s potential as therapeutic target for lapatinib insensitive BC patients. One of the most commonly used commercially available reagents for VDR targeted therapy is Calcitriol, the active hormonal form of Vitamin D_3_ [73]. It primarily functions by binding to and activating VDR leading to signalling and transcriptomic changes [73]. However, the effect of calcitriol depends on dose, frequency and time lapsed following treatment. For instance, low levels of calcitriol have been shown to possess pro-tumorigenic effects, whereas high doses of calcitriol, especially when applied in pulses, were shown to have anti-proliferative and anti-tumorigenic effects in several types of cancer cells [74–76]. Although at the protein level calcitriol activates VDR signalling by binding to it, it was also shown to reduce the mRNA expression level of VDR [77–79]. Recently, combinations of calcitriol and antiestrogen or gefitinib (a tyrosine kinase inhibitor), respectively, were found to reduce proliferation and increase apoptosis of BC cells more effectively than any of these compounds alone [80, 81].

We investigated whether the combination of calcitriol and lapatinib can be used as an effective treatment strategy for lapatinib insensitive BC patients. We selected four cell lines for our experiment. Two of these, MDA-MB-453 and JIMT-1 are HER2 positive but innately insensitive to lapatinib. Whereas HCC 1954-L cell lines, which was developed by exposing HER2 positive HCC 1954 cells to lapatinib for a long period of time, has acquired resistance to lapatinib. The clinico-pathological subtypes and the expression levels of HER2, EGFR and VDR which are targets of lapatinib and calcitriol are shown in Table 3.

**Table 3:** Clinico-pathological subtypes of and receptor expressions in MDA-MB-453 and JIMT1 cell lines.

Cell line	Clinico pathological subtype	HER2 (percentile)	EGFR (percentile)	VDR (percentile)
MDA-MB-453	Triple negative[82], HER2 positive [83]	97.50	79.03	76.18
JIMT-1	HER2 positive	97.30	2.90	95.33
HCC 1954	HER2 positive	99.21	76.20	87.92

The receptor expression data were collected from the GDSC database [34]. The expression data are shown in percentile, here *X* percentile means *X* percent of all cell lines in the GDSC database [34] had equal or less expression than the above cell lines.

Among the innately lapatinib insensitive cell lines, clinico-pathological subtype of MDA-MB-453 is fiercely debated in literature, while some suggest it is a triple negative BC cell with no or low HER2 expression [82], others reports it to be HER2 positive with overexpressed HER2 [83]. We compared HER2 expression in MDA-MB-453 cells with other cancer cell lines in GDSC database [34] and found that HER2 has higher mRNA expression in MDA-MB-453 than ~97.5% of all (1018) cell lines in this database. JIMT-1 is known to be HER2 positive [84] which is corroborated by the GDSC data [34] (Table 3). While EGFR is over expressed in MDA-MB-453, it is significantly under-expressed in JIMT-1 (Table 3). VDR has above average expressions in MDA-MB-453 and JIMT-1 cell lines and have higher expression in JIMT1 than in MDA-MB-453 according to the GDSC database. However, in our qRT-PCR analysis VDR has higher expression in MDA-MB-453 than in JIMT-1 cells (Figure A in S1 File). Despite overexpressing at least one of HER2 or EGFR neither of these cell lines respond to lapatinib. Following 16 hours of treatment with 1 μM lapatinib neither cell-line showed a significant change in VDR gene expression (Fig 4F), reflecting the same behaviour as the lapatinib insensitive T47D and MDA-MB-468 cell lines, which were used to generate the time course gene expression data.

HCC1954, which was used to develop HCC 1954-L with acquired lapatinib resistace, is known to be HER2 positive and has overexpressed HER2, EGFR, and VDR (Table 3).

The cells were treated with calcitriol (6 *μM* for MDA-MB-453, JIMT-1, HCC 1954 and 500nM for HCC 1954-L) and lapatinib (0.25 *μM*, 0.5 *μM*, 1.0 *μM* for all four cell lines), individually and in combination (calcitriol + lapatinib) for 5 days before measuring cell proliferation. In MDA-MB-453, JIMT-1, HCC1954 and HCC1954-L cells calcitriol alone resulted in 19%, 11%, 21% and 30% growth inhibition respectively (Fig 4G–4J), whereas, different doses of lapatinib inhibited the growth of MDA-MB-453, JIMT-1, HCC1954 and HCC1954-L cells by 10–19%, 1–5%, 50–64% and 25–44% respectively (Fig 4G–4J). However, the combination of calcitriol + lapatinib resulted in 36–38%, 13–17% and 43–52%, 49–70% growth inhibition in MDA-MB-453, JIMT-1, HCC1954 and HCC1954-L cells respectively depending on the doses of lapatinib (Fig 4G–4J). Thus, among the innately insensitive cells, treatment with lapatinib and calcitriol showed a significant additive effect in MDA-MB-453 but not in JIMT-1 cells. The lower expression levels of VDR expression in the JIMT-1 cells compared to MDA-MB-453 cells may plausibly explain why these cells are less sensitive to calcitriol treatment.

On the other hand, among the isogenic HCC1954 and HCC1954-L cells, only the latter displayed an additive effect of the lapatinib and calcitriol combination (Fig 4I and 4J). Based on qRT-PCR analysis, VDR mRNA expression is increased in the HCC1954-L cells (1.7 fold) (Figure B in S1 File), which may contribute to benefit observed with calcitriol treatment.

## 4 Discussion

The rapid advent of omics technology made it possible to investigate the molecular networks of living cells in unprecedented detail. Consequently, it is now possible to track the behaviours of thousands of genes simultaneously over a period of time. However, identifying genes which show different temporal behaviour under different experimental conditions is not straightforward. Here, we developed a statistical tool that addresses this question and applied it to study the gene expression changes associated with lapatinib resistance in BC cells. Using existing datasets, our study identified several potential biomarkers of lapatinib resistance in BC, some of which were experimentally validated.

We identified more than 500 genes as potential lapatinib resistance markers, many of which can also be potential targets for treating lapatinib and, more generally, HER2-targeted TKI resistant BC cells. One such target identified in this study is VDR. We experimentally verified whether VDR can be a viable target for combination therapy for treating HER2 positive lapatinib resistant (innate and acquired) BC patients. We selected two cell lines for the investigation of innate insensitivity, MDM-MB-453 where both HER2 and VDR are relatively highly expressed, and JIMT-1 where HER2 is highly expressed but VDR has significantly lower mRNA expression compared to MDA-MB-453. The combination of lapatinib and calcitriol inhibited proliferation more effectively than individual treatment by either reagents in MDA-MB-453, but not in JIMT-1. In the acquired resistance setting we used the HCC1954-L cells which were developed by exposing HCC1954 cells to lapatinib for 6 months. The combination of calcitriol and lapatinib inhibited proliferation of the lapatinib resistant HCC1954-L more effectively than either agent alone. These results suggest that the combination of calcitriol and lapatinib can be a potential combination therapy regimen for patients who express HER2 and VDR but do not respond to lapatinib treatment. However, these are preliminary results and need further experimental validation in a larger panel of cell lines and animal models to assess the clinical viability of this drug combination.

Drug resistance is a complex process which involves dynamic adaptations of the interplay between signalling and transcriptional networks of cancer cells induced by drug treatments. A system level understanding of transcriptional and signalling networks is required to design therapeutic strategies targeted at overcoming such resistance. Such insight can be gained by reconstructing quantitative models of transcriptional and signalling network from experimental data using network reconstruction methods [50, 85–90]. The reconstructed models can then be used to simulate the effects of drug treatments on signalling and transcriptional networks of drug resistant cancer cells. We are currently pursuing this avenue to identify potent combination therapy regimens for treating BC patients.

## Supporting information

S1 File Supplementary information for this manuscript. Contains supplementary Tables A-C and supplementary Figures A,B.
(DOCX)

## References

[1] Stewart BW, Wild CP. World Cancer Report 2014: World Health Organization; 2014.

[2] O'Brien NA, Browne BC, Chow L, Wang Y, Ginther C, Arboleda J, et al Activated phosphoinositide 3-kinase/AKT signaling confers resistance to trastuzumab but not lapatinib. Mol Cancer Ther. 2010;9(6):1489–502. 10.1158/1535-7163.MCT-09-1171 20501798

[3] Slamon DJ, Clark GM, Wong SG, Levin WJ, Ullrich A, McGuire WL. Human breast cancer: correlation of relapse and survival with amplification of the HER-2/neu oncogene. Science. 1987;235(4785):177–82. 3798106 10.1126/science.3798106

[4] Gajria D, Chandarlapaty S. HER2-amplified breast cancer: mechanisms of trastuzumab resistance and novel targeted therapies. Expert Rev Anticancer Ther. 2011;11(2):263–75. 10.1586/era.10.226 21342044 PMC3092522

[5] Singh JC, Jhaveri K, Esteva FJ. HER2-positive advanced breast cancer: optimizing patient outcomes and opportunities for drug development. Br J Cancer. 2014;111(10):1888–98. 10.1038/bjc.2014.388 25025958 PMC4229628

[6] Burris HA 3rd, Hurwitz HI, Dees EC, Dowlati A, Blackwell KL, O'Neil B, et al Phase I safety, pharmacokinetics, and clinical activity study of lapatinib (GW572016), a reversible dual inhibitor of epidermal growth factor receptor tyrosine kinases, in heavily pretreated patients with metastatic carcinomas. J Clin Oncol. 2005;23(23):5305–13. 10.1200/JCO.2005.16.584 15955900

[7] Rusnak DW, Lackey K, Affleck K, Wood ER, Alligood KJ, Rhodes N, et al The effects of the novel, reversible epidermal growth factor receptor/ErbB-2 tyrosine kinase inhibitor, GW2016, on the growth of human normal and tumor-derived cell lines in vitro and in vivo. Mol Cancer Ther. 2001;1(2):85–94. 12467226

[8] Geyer CE, Forster J, Lindquist D, Chan S, Romieu CG, Pienkowski T, et al Lapatinib plus capecitabine for HER2-positive advanced breast cancer. N Engl J Med. 2006;355(26):2733–43. 10.1056/NEJMoa064320 17192538

[9] Konecny GE, Pegram MD, Venkatesan N, Finn R, Yang G, Rahmeh M, et al Activity of the dual kinase inhibitor lapatinib (GW572016) against HER-2-overexpressing and trastuzumab-treated breast cancer cells. Cancer Res. 2006;66(3):1630–9. 10.1158/0008-5472.CAN-05-1182 16452222

[10] Blackwell KL, Burstein HJ, Storniolo AM, Rugo H, Sledge G, Koehler M, et al Randomized study of Lapatinib alone or in combination with trastuzumab in women with ErbB2-positive, trastuzumab-refractory metastatic breast cancer. J Clin Oncol. 2010;28(7):1124–30. 10.1200/JCO.2008.21.4437 20124187

[11] Gomez HL, Doval DC, Chavez MA, Ang PC, Aziz Z, Nag S, et al Efficacy and safety of lapatinib as first-line therapy for ErbB2-amplified locally advanced or metastatic breast cancer. J Clin Oncol. 2008;26(18):2999–3005. 10.1200/JCO.2007.14.0590 18458039

[12] Hegde PS, Rusnak D, Bertiaux M, Alligood K, Strum J, Gagnon R, et al Delineation of molecular mechanisms of sensitivity to lapatinib in breast cancer cell lines using global gene expression profiles. Mol Cancer Ther. 2007;6(5):1629–40. 10.1158/1535-7163.MCT-05-0399 17513611

[13] Grigorov MG. Analysis of time course Omics datasets. Methods Mol Biol. 2011;719:153–72. 10.1007/978-1-61779-027-0_7 21370083

[14] Hejblum BP, Skinner J, Thiebaut R. Time-Course Gene Set Analysis for Longitudinal Gene Expression Data. PLoS Comput Biol. 2015;11(6):e1004310 10.1371/journal.pcbi.1004310 26111374 PMC4482329

[15] Ganesh SK, Joo J, Skelding K, Mehta L, Zheng G, O'Neill K, et al Time course analysis of gene expression identifies multiple genes with differential expression in patients with in-stent restenosis. BMC Med Genomics. 2011;4:20 10.1186/1755-8794-4-20 21356094 PMC3053213

[16] Hensman J, Lawrence ND, Rattray M. Hierarchical Bayesian modelling of gene expression time series across irregularly sampled replicates and clusters. BMC Bioinformatics. 2013;14:252 10.1186/1471-2105-14-252 23962281 PMC3766667

[17] Kalaitzis AA, Lawrence ND. A simple approach to ranking differentially expressed gene expression time courses through Gaussian process regression. BMC Bioinformatics. 2011;12:180 10.1186/1471-2105-12-180 21599902 PMC3116489

[18] Kayano M, Matsui H, Yamaguchi R, Imoto S, Miyano S. Gene set differential analysis of time course expression profiles via sparse estimation in functional logistic model with application to time-dependent biomarker detection. Biostatistics. 2015. 10.1093/biostatistics/kxv037 26420796

[19] Kim J, Ogden RT, Kim H. A method to identify differential expression profiles of time-course gene data with Fourier transformation. BMC Bioinformatics. 2013;14:310 10.1186/1471-2105-14-310 24134721 PMC4015127

[20] Liang Y, Tayo B, Cai X, Kelemen A. Differential and trajectory methods for time course gene expression data. Bioinformatics. 2005;21(13):3009–16. 10.1093/bioinformatics/bti465 15886280 PMC2574001

[21] Topa H, Jonas A, Kofler R, Kosiol C, Honkela A. Gaussian process test for high-throughput sequencing time series: application to experimental evolution. Bioinformatics. 2015;31(11):1762–70. 10.1093/bioinformatics/btv014 25614471 PMC4443671

[22] Rasmussen CE, Williams CKI. Gaussian Processes for Machine Learning: University Press Group Limited; 2006.

[23] Donders ART, van der Heijden GJMG, Stijnen T, Moons KGM. Review: A gentle introduction to imputation of missing values. Journal of Clinical Epidemiology. 59(10):1087–91. 10.1016/j.jclinepi.2006.01.014 16980149

[24] Charlebois DA. Effect and evolution of gene expression noise on the fitness landscape. Physical Review E. 2015;92(2):022713. 10.1103/PhysRevE.92.022713 26382438

[25] Maheshri N, O'Shea EK. Living with noisy genes: how cells function reliably with inherent variability in gene expression. Annu Rev Biophys Biomol Struct. 2007;36:413–34. 10.1146/annurev.biophys.36.040306.132705 17477840

[26] McDermott M, Eustace AJ, Busschots S, Breen L, Crown J, Clynes M, et al In vitro Development of Chemotherapy and Targeted Therapy Drug-Resistant Cancer Cell Lines: A Practical Guide with Case Studies. Frontiers in Oncology. 2014;4:40 10.3389/fonc.2014.00040 24639951 PMC3944788

[27] Livak KJ, Schmittgen TD. Analysis of relative gene expression data using real-time quantitative PCR and the 2(-Delta Delta C(T)) Method. Methods. 2001;25(4):402–8. 10.1006/meth.2001.1262 11846609

[28] Martin A, Clynes M. Acid phosphatase: endpoint for in vitro toxicity tests. In Vitro Cell Dev Biol. 1991;27A(3 Pt 1):183–4. 2033016 10.1007/BF02630912

[29] Uhlenbeck GE, Ornstein LS. On the Theory of the Brownian Motion. Physical Review. 1930;36(5):823–41.

[30] Rohlfs RV, Harrigan P, Nielsen R. Modeling gene expression evolution with an extended Ornstein-Uhlenbeck process accounting for within-species variation. Mol Biol Evol. 2014;31(1):201–11. 10.1093/molbev/mst190 24113538 PMC3879452

[31] Hajian-Tilaki K. Receiver Operating Characteristic (ROC) Curve Analysis for Medical Diagnostic Test Evaluation. Caspian J Intern Med. 2013;4(2):627–35. 24009950 PMC3755824

[32] Holliday DL, Speirs V. Choosing the right cell line for breast cancer research. Breast Cancer Research. 2011;13(4):215 10.1186/bcr2889 21884641 PMC3236329

[33] Henjes F, Bender C, von der Heyde S, Braun L, Mannsperger HA, Schmidt C, et al Strong EGFR signaling in cell line models of ERBB2-amplified breast cancer attenuates response towards ERBB2-targeting drugs. Oncogenesis. 2012;1:e16 http://www.nature.com/oncsis/journal/v1/n7/suppinfo/oncsis201216s1.html. 10.1038/oncsis.2012.16 23552733 PMC3412653

[34] Yang W, Soares J, Greninger P, Edelman EJ, Lightfoot H, Forbes S, et al Genomics of Drug Sensitivity in Cancer (GDSC): a resource for therapeutic biomarker discovery in cancer cells. Nucleic Acids Res. 2013;41(D1):D955–D61. 23180760 10.1093/nar/gks1111 PMC3531057

[35] Huang da W, Sherman BT, Lempicki RA. Systematic and integrative analysis of large gene lists using DAVID bioinformatics resources. Nat Protoc. 2009;4(1):44–57. 10.1038/nprot.2008.211 19131956

[36] Huang da W, Sherman BT, Lempicki RA. Bioinformatics enrichment tools: paths toward the comprehensive functional analysis of large gene lists. Nucleic Acids Res. 2009;37(1):1–13. 10.1093/nar/gkn923 19033363 PMC2615629

[37] Supek F, Bosnjak M, Skunca N, Smuc T. REVIGO summarizes and visualizes long lists of gene ontology terms. PLoS One. 2011;6(7):e21800 10.1371/journal.pone.0021800 21789182 PMC3138752

[38] Kanehisa M, Goto S, Sato Y, Kawashima M, Furumichi M, Tanabe M. Data, information, knowledge and principle: back to metabolism in KEGG. Nucleic Acids Res. 2014;42(Database issue):D199–205. 10.1093/nar/gkt1076 24214961 PMC3965122

[39] Mi H, Thomas P. PANTHER pathway: an ontology-based pathway database coupled with data analysis tools. Methods Mol Biol. 2009;563:123–40. 10.1007/978-1-60761-175-2_7 19597783 PMC6608593

[40] Batut P, Dobin A, Plessy C, Carninci P, Gingeras TR. High-fidelity promoter profiling reveals widespread alternative promoter usage and transposon-driven developmental gene expression. Genome Res. 2013;23(1):169–80. 10.1101/gr.139618.112 22936248 PMC3530677

[41] Consortium EP. An integrated encyclopedia of DNA elements in the human genome. Nature. 2012;489(7414):57–74. 10.1038/nature11247 22955616 PMC3439153

[42] Lambert N, Robertson A, Jangi M, McGeary S, Sharp PA, Burge CB. RNA Bind-n-Seq: quantitative assessment of the sequence and structural binding specificity of RNA binding proteins. Mol Cell. 2014;54(5):887–900. 10.1016/j.molcel.2014.04.016 24837674 PMC4142047

[43] Bovolenta LA, Acencio ML, Lemke N. HTRIdb: an open-access database for experimentally verified human transcriptional regulation interactions. BMC Genomics. 2012;13:405 10.1186/1471-2164-13-405 22900683 PMC3472291

[44] Huang R, Faratian D, Sims AH, Wilson D, Thomas JS, Harrison DJ, et al Increased STAT1 signaling in endocrine-resistant breast cancer. PLoS One. 2014;9(4):e94226 10.1371/journal.pone.0094226 24728078 PMC3984130

[45] Hannesdottir L, Tymoszuk P, Parajuli N, Wasmer MH, Philipp S, Daschil N, et al Lapatinib and doxorubicin enhance the Stat1-dependent antitumor immune response. Eur J Immunol. 2013;43(10):2718–29. 10.1002/eji.201242505 23843024

[46] Lee YY, Kim HP, Kang MJ, Cho BK, Han SW, Kim TY, et al Phosphoproteomic analysis identifies activated MET-axis PI3K/AKT and MAPK/ERK in lapatinib-resistant cancer cell line. Exp Mol Med. 2013;45:e64 10.1038/emm.2013.115 24263233 PMC3849569

[47] Fiorito E, Katika MR, Hurtado A. Cooperating transcription factors mediate the function of estrogen receptor. Chromosoma. 2013;122(1–2):1–12. 10.1007/s00412-012-0392-7 23192763 PMC3608891

[48] Gee JM, Eloranta JJ, Ibbitt JC, Robertson JF, Ellis IO, Williams T, et al Overexpression of TFAP2C in invasive breast cancer correlates with a poorer response to anti-hormone therapy and reduced patient survival. J Pathol. 2009;217(1):32–41. 10.1002/path.2430 18825690

[49] Rosenfeldt MT, Bell LA, Long JS, O'Prey J, Nixon C, Roberts F, et al E2F1 drives chemotherapeutic drug resistance via ABCG2. Oncogene. 2014;33(32):4164–72. 10.1038/onc.2013.470 24276245

[50] Petralia F, Wang P, Yang J, Tu Z. Integrative random forest for gene regulatory network inference. Bioinformatics. 2015;31(12):i197–i205. 10.1093/bioinformatics/btv268 26072483 PMC4542785

[51] Yan LH, Wei WY, Cao WL, Zhang XS, Xie YB, Xiao Q. Overexpression of E2F1 in human gastric carcinoma is involved in anti-cancer drug resistance. BMC Cancer. 2014;14:904 10.1186/1471-2407-14-904 25466554 PMC4258940

[52] Bryan EJ, Jokubaitis VJ, Chamberlain NL, Baxter SW, Dawson E, Choong DY, et al Mutation analysis of EP300 in colon, breast and ovarian carcinomas. Int J Cancer. 2002;102(2):137–41. 10.1002/ijc.10682 12385008

[53] Docquier F, Farrar D, D'Arcy V, Chernukhin I, Robinson AF, Loukinov D, et al Heightened expression of CTCF in breast cancer cells is associated with resistance to apoptosis. Cancer Res. 2005;65(12):5112–22. 10.1158/0008-5472.CAN-03-3498 15958555

[54] McKay JD, McCullough ML, Ziegler RG, Kraft P, Saltzman BS, Riboli E, et al Vitamin D Receptor Polymorphisms and Breast Cancer Risk: Results from the National Cancer Institute Breast and Prostate Cancer Cohort Consortium. Cancer Epidemiology Biomarkers & Prevention. 2009;18(1):297–305. 10.1158/1055-9965.EPI-08-0539 19124512

[55] Szklarczyk D, Franceschini A, Wyder S, Forslund K, Heller D, Huerta-Cepas J, et al STRING v10: protein-protein interaction networks, integrated over the tree of life. Nucleic Acids Res. 2015;43(Database issue):D447–52. 10.1093/nar/gku1003 25352553 PMC4383874

[56] McDermott MS, Browne BC, Conlon NT, O'Brien NA, Slamon DJ, Henry M, et al PP2A inhibition overcomes acquired resistance to HER2 targeted therapy. Mol Cancer. 2014;13:157 10.1186/1476-4598-13-157 24958351 PMC4230643

[57] O'Neill F, Madden S, Aherne S, Clynes M, Crown J, Doolan P, et al Gene expression changes as markers of early lapatinib response in a panel of breast cancer cell lines. Molecular Cancer. 2012;11(1):41. 22709873 10.1186/1476-4598-11-41 PMC3439312

[58] Schefe JH, Lehmann KE, Buschmann IR, Unger T, Funke-Kaiser H. Quantitative real-time RT-PCR data analysis: current concepts and the novel "gene expression's CT difference" formula. J Mol Med (Berl). 2006;84(11):901–10. 16972087 10.1007/s00109-006-0097-6

[59] Yuan JS, Reed A, Chen F, Stewart CN Jr. Statistical analysis of real-time PCR data. BMC Bioinformatics. 2006;7:85 10.1186/1471-2105-7-85 16504059 PMC1395339

[60] Creighton CJ. Widespread Molecular Patterns Associated with Drug Sensitivity in Breast Cancer Cell Lines, with Implications for Human Tumors. PLoS ONE. 2013;8(12):e71158 10.1371/journal.pone.0071158 24386073 PMC3873899

[61] Mayer IA, Arteaga CL. Does Lapatinib Work against HER2-negative Breast Cancers? Clinical cancer research: an official journal of the American Association for Cancer Research. 2010;16(5):1355–7. 20179241 10.1158/1078-0432.CCR-09-3223 PMC3448974

[62] D’Amato V, Raimondo L, Formisano L, Giuliano M, De Placido S, Rosa R, et al Mechanisms of lapatinib resistance in HER2-driven breast cancer. Cancer Treatment Reviews. 2015;41(10):877–83. 10.1016/j.ctrv.2015.08.001. 26276735

[63] Szász AM, Lánczky A, Nagy Á, Förster S, Hark K, Green JE, et al Cross-validation of survival associated biomarkers in gastric cancer using transcriptomic data of 1,065 patients 2016. 10.18632/oncotarget.10337 PMC5226511 27384994

[64] Kim S, Kim DH, Jung W-H, Koo JS. Succinate dehydrogenase expression in breast cancer. SpringerPlus. 2013;2(1):299 10.1186/2193-1801-2-299 23888270 PMC3710570

[65] Pons DG, Nadal-Serrano M, Torrens-Mas M, Valle A, Oliver J, Roca P. UCP2 inhibition sensitizes breast cancer cells to therapeutic agents by increasing oxidative stress. Free Radical Biology and Medicine. 2015;86:67–77. 10.1016/j.freeradbiomed.2015.04.032. 25960046

[66] van der Watt PJ, Ngarande E, Leaner VD. Overexpression of Kpnβ1 and Kpnα2 importin proteins in cancer derives from deregulated E2F activity. PLoS One. 2011;6(11):e27723 10.1371/journal.pone.0027723 22125623 PMC3220698

[67] Okolicsanyi RK, Buffiere A, Jacinto JME, Chacon-Cortes D, Chambers SK, Youl PH, et al Association of heparan sulfate proteoglycans SDC1 and SDC4 polymorphisms with breast cancer in an Australian Caucasian population. Tumor Biology. 2015;36(3):1731–8. 10.1007/s13277-014-2774-3 25361632

[68] Zhao P, Nairn AV, Hester S, Moremen KW, O'Regan RM, Oprea G, et al Proteomic Identification of Glycosylphosphatidylinositol Anchor-dependent Membrane Proteins Elevated in Breast Carcinoma. The Journal of Biological Chemistry. 2012;287(30):25230–40. 10.1074/jbc.M112.339465 22654114 PMC3408190

[69] Shapiro IM, Cheng AW, Flytzanis NC, Balsamo M, Condeelis JS, Oktay MH, et al An EMT–Driven Alternative Splicing Program Occurs in Human Breast Cancer and Modulates Cellular Phenotype. PLOS Genetics. 2011;7(8):e1002218 10.1371/journal.pgen.1002218 21876675 PMC3158048

[70] Mangelsdorf DJ, Thummel C, Beato M, Herrlich P, SchÃ¼tz Gn, Umesono K, et al The nuclear receptor superfamily: The second decade. Cell. 1995;83(6):835–9. 8521507 10.1016/0092-8674(95)90199-x PMC6159888

[71] zur Hausen H. The role of microRNAs in human cancer. International Journal of Cancer. 2008;122(5):ix–ix.

[72] Kurozumi S, Yamaguchi Y, Kurosumi M, Ohira M, Matsumoto H, Horiguchi J. Recent trends in microRNA research into breast cancer with particular focus on the associations between microRNAs and intrinsic subtypes. J Hum Genet. 2016. 10.1038/jhg.2016.89 27439682

[73] Feldman D, Krishnan AV, Swami S, Giovannucci E, Feldman BJ. The role of vitamin D in reducing cancer risk and progression. Nat Rev Cancer. 2014;14(5):342–57. 10.1038/nrc3691 24705652

[74] Beer TM, Myrthue A. Calcitriol in cancer treatment: From the lab to the clinic. Molecular Cancer Therapeutics. 2004;3(3):373–81. 15026558

[75] Brawer MK. Recent Progress in the Treatment of Advanced Prostate Cancer With Intermittent Dose-Intense Calcitriol (DN-101). Reviews in Urology. 2007;9(1):1–8. 17396166 PMC1831525

[76] Muindi JR, Peng Y, Potter DM, Hershberger PA, Tauch JS, Capozzoli MJ, et al Pharmacokinetics of high-dose oral calcitriol: Results from a phase 1 trial of calcitriol and paclitaxel. Clinical Pharmacology & Therapeutics. 2002;72(6):648–59. 12496746 10.1067/mcp.2002.129305

[77] Beer TM, Myrthue A, Garzotto M, O'Hara M F, Chin R, Lowe BA, et al Randomized study of high-dose pulse calcitriol or placebo prior to radical prostatectomy. Cancer Epidemiol Biomarkers Prev. 2004;13(12):2225–32. 15598784

[78] Patel SR, Ke HQ, Hsu CH. Regulation of calcitriol receptor and its mRNA in normal and renal failure rats. Kidney international. 1994;45(4):1020–7. 8007571 10.1038/ki.1994.138

[79] Thill M, Cordes T, Hoellen F, Becker S, Dittmer C, Kuemmel S, et al Influence of calcitriol on prostaglandin-and vitamin D-metabolising enzymes in benign and malignant breast cell lines. Anticancer research. 2012;32(1):359–65. 22213327

[80] Santos-Martínez N, Díaz L, Ordaz-Rosado D, García-Quiroz J, Barrera D, Avila E, et al Calcitriol restores antiestrogen responsiveness in estrogen receptor negative breast cancer cells: A potential new therapeutic approach. BMC cancer. 2014;14(1):230. 24678876 10.1186/1471-2407-14-230 PMC3972996

[81] Segovia-Mendoza M, Díaz L, González-González ME, Martínez-Reza I, García-Quiroz J, Prado-Garcia H, et al Calcitriol and its analogues enhance the antiproliferative activity of gefitinib in breast cancer cells. The Journal of steroid biochemistry and molecular biology. 2015;148:122–31. 10.1016/j.jsbmb.2014.12.006 25510900

[82] Holliday DL, Speirs V. Choosing the right cell line for breast cancer research. Breast Cancer Research. 2011;13(4):1. 10.1186/bcr2889 PMC3236329 21884641

[83] Neve RM, Chin K, Fridlyand J, Yeh J, Baehner FL, Fevr T, et al A collection of breast cancer cell lines for the study of functionally distinct cancer subtypes. Cancer Cell. 2006;10(6):515–27. 10.1016/j.ccr.2006.10.008. 17157791 PMC2730521

[84] Park SH, Ito K, Olcott W, Katsyv I, Halstead-Nussloch G, Irie HY. PTK6 inhibition promotes apoptosis of Lapatinib-resistant Her2(+) breast cancer cells by inducing Bim. Breast Cancer Research: BCR. 2015;17(1):86. 26084280 10.1186/s13058-015-0594-z PMC4496943

[85] Wang YXR, Huang H. Review on statistical methods for gene network reconstruction using expression data. Journal of Theoretical Biology. 2014;362:53–61. 10.1016/j.jtbi.2014.03.040. 24726980

[86] Santra T. A bayesian framework that integrates heterogeneous data for inferring gene regulatory networks. Frontiers in bioengineering and biotechnology. 2014;2. 10.3389/fbioe.2014.00013 PMC4126456 25152886

[87] Santra T, Kolch W, Kholodenko BN. Integrating Bayesian variable selection with Modular Response Analysis to infer biochemical network topology. BMC Systems Biology. 2013;7(1):1–19. 23829771 10.1186/1752-0509-7-57 PMC3726398

[88] Iglesias-Martinez LF, Kolch W, Santra T. BGRMI: A method for inferring gene regulatory networks from time-course gene expression data and its application in breast cancer research. Scientific Reports. 2016;6:37140 10.1038/srep37140 27876826 PMC5120305

[89] Santra T, Kholodenko B, Kolch W. An integrated bayesian framework for identifying phosphorylation networks in stimulated cells. Advances in Systems Biology. 2012;736:59–80. 10.1007/978-1-4419-7210-1_3 22161322

[90] Halasz M, Kholodenko BN, Kolch W, Santra T. Integrating network reconstruction with mechanistic modeling to predict cancer therapies. Sci Signal. 2016;9(455):ra114–ra. 10.1126/scisignal.aae0535 27879396

