# Supplementary material for: Identification of potential new treatment response markers and therapeutic targets using a Gaussian process-based method in lapatinib insensitive breast cancer models
Source: PLoS One. 2017 May 8;12(5):e0177058. doi: 10.1371/journal.pone.0177058 (PMC5421758; doi:10.1371/journal.pone.0177058)
Supplement: S1 File — Contains supplementary Tables A-C and supplementary Figures A,B. (DOCX) [file pone.0177058.s001.docx]

# Supplementary File S1: Supplementary information for manuscript titled “Identification of Potential New Treatment Response Markers and Therapeutic Targets Using a Gaussian Process-based Method in Lapatinib Insensitive Breast Cancer Models”

Tapesh Santra^1+^*, Sandra Roche^2+^, Neil Conlon, Norma O’Donovan^2^, John Crown^2,3^, Robert O’Connor^2^, Walter Kolch^1,4,5^

^1^Systems Biology Ireland, University College Dublin, Belfield, Dublin-4, Ireland

^2^National Institute for Cellular Biotechnology, Dublin City University, Dublin 9, Ireland

^3^Department of Medical Oncology, St Vincent’s University Hospital, Elm Park, Dublin 4, Ireland

^4^Conway Institute of Biomolecular and Biomedical Research, University College Dublin, Belfield, Dublin, Ireland

^5^School of Medicine, University College Dublin, Belfield, Dublin, Ireland

*****Corresponding author

Email: tapesh.santra@ucd.ie

+ Authors contributed equally

**Table A:** Lapatinib insensitivity markers identified by GEAGP.

| STAT1 | PSMA7 | GAS7 | PCBP2 | LLGL1 | ISCU | DAG1 | GPSM3 | SLTM | NCAPG2 |
| --- | --- | --- | --- | --- | --- | --- | --- | --- | --- |
| BAT1 | COX8A | HSD17B10 | CLTA | CLC | UBE2V2 | ATP2B4 | ZCWPW2 | HSD17B12 | CCDC21 |
| USP22 | ITGB5 | PPP2R2A | PIGQ | PEX10 | UFD1L | KIDINS220 | F13B | SUCLG1 | TRPV1 |
| HINT1 | EIF2S1 | CSNK1E | MFNG | CHRNA5 | IRS2 | XPO7 | EXOC7 | KLHDC2 | LSM14B |
| PLD1 | RAB5C | HD | ADRBK2 | MIA | C3orf63 | EIF1 | IFI6 | IQWD1 | TDP1 |
| E2F1 | NUDC | AP3S2 | PKNOX1 | CNR2 | AMACR | MAPK1 | IGHA1 | C19orf53 | ZNF432 |
| CHST3 | ARCN1 | NOTCH2 | PSMC1 | FOXN2 | CCBL2 | OGT | IGHG1 | STUB1 | RUFY2 |
| FAM89B | AMD1 | POLS | VDR | MAP3K7 | TXNDC1 | VPS13D | IL8 | BAZ1A | KPTN |
| DMWD | PSMD1 | SNRPB2 | ZNF518 | CPNE1 | PRUNE | YIPF6 | SIX6 | RBM47 | CUZD1 |
| B3GAT3 | CREG1 | CTCF | FUSIP1 | NFRKB | KIAA0947 | USP24 | RAC1 | NMD3 | GDPD2 |
| SGSM2 | PCNA | PITPNB | TMEM187 | ATP4A | DCLRE1A | EFHA1 | SCNN1A | XTP3TPA | FAM48A |
| SBF1 | DAB2 | DHFR | TMED2 | GNG5 | ANXA7 | C14orf32 | MGAT5 | UNC119 | VAX2 |
| WDTC1 | SDC1 | CHMP2B | ProSAPiP1 | SH2D2A | PUF60 | STAT5B | WDR45 | ANKRD10 | C20orf30 |
| FAM65A | ENC1 | SLC30A9 | DST | SMTN | DHTKD1 | DAZAP2 | GTF2IRD2 | MLPH | MCTP2 |
| CXXC1 | SF3A1 | CSDE1 | LYPLA3 | PIP5K1A | CFLAR | HNRNPC | APOBEC3G | NDUFB4 | ZNF614 |
| LOC400451 | PPP2CB | MAP2K1 | ZNF142 | SNUPN | ZNF410 | PTPLB | PPP2CA | GTPBP4 | DNAH3 |
| COL8A2 | UBAP2L | PIK3R3 | OSTF1 | CD46 | JTV1 | TLN2 | RIMBP2 | NUP54 | TRIM69 |
| LDLRAP1 | NQO1 | SS18 | PPIC | RAB1A | TAF5 | LARP4 | SNRPA1 | POLR1D | PPA2 |
| ZGPAT | SQSTM1 | TCEB3 | PPP2R5B | RNF10 | SPAG1 | SFRS12 | MPHOSPH9 | MKL2 | MMP27 |
| EXOSC4 | VBP1 | MMP14 | TRA2A | GUCY2D | EIF6 | JMJD6 | DMXL2 | GALNT7 | PRO1768 |
| ING4 | SUPT5H | POP4 | FOXA1 | MAEA | TP53AP1 | SUB1 | GSTA1 | COMMD8 | FRS2 |
| AP2M1 | AAMP | MOBKL3 | ICAM2 | CSN2 | TRIM33 | LOC137886 | LOC23117 | METT11D1 | NEUROD4 |
| CALM3 | TOMM70A | ARFGEF1 | WIPI2 | ACRV1 | MPZ | ZDHHC17 | LOC388237 | U2AF2 | CTLA4 |
| YWHAZ | RPA1 | SGSM3 | IL11RA | GML | TPD52L1 | FANCI | LOC440350 | APH1A | MCHR1 |
| HDLBP | SAR1A | MTSS1 | PRKAR2A | CCR8 | RASGRF1 | NEDD4 | MAGEC2 | DNAJC15 | FGF17 |
| GABARAP | SMC1A | BMS1 | PLK4 | GAST | MPZL1 | ZRF1 | BTRC | C16orf61 | MRLC2 |
| S100A11 | ALDH9A1 | CDYL | BCL3 | INPP4A | ATG5 | FBXL14 | KLC1 | TMEM33 | ATP6V1H |
| UBE2D3 | GPAA1 | KIAA0040 | MLLT3 | TBPL1 | MASP1 | PIBF1 | TBCB | C21orf66 | CCPG1 |
| DDX24 | PRDX3 | SRPK2 | LYPD3 | ABCC9 | ARL17P1 | DKFZp586I1420 | SEC11A | CHMP4A | LYK5 |
| DYNLL1 | CYB5B | APRT | FGFBP1 | PTP4A2 | LYN | EP300 | TAOK1 | MRPS33 | DKFZP564O0523 |
| FKBP1A | CDC123 | RFK | NCAPH2 | HADHA | SIP1 | ZNF710 | HMG1L1 | ATPIF1 | RECQL5 |
| RPL10 | ELAVL1 | CLK2 | CCR1 | TTC3 | PTGER3 | MARS | HMGB1 | HYPK | NPM1 |
| PTP4A1 | MAR06 | ARHGEF9 | ACCN2 | HMGN2 | IQGAP1 | CALM1 | CPNE9 | PDLIM4 | MRPL34 |
| PGK1 | EIF1B | ADNP2 | IL1RAP | NONO | EFS | COX5B | PLCG1 | PRR14 | GPATCH4 |
| CTNNA1 | SFRS1 | COL5A1 | POU2AF1 | NDUFV1 | MGLL | ATP5A1 | SH3TC1 | NOL9 | TNS1 |
| FAM120A | SAFB | NDUFB3 | TFAP2C | FLOT1 | CLTB | ASCL1 | EPHA5 | TMEM38B | SLC2A3P1 |
| ECH1 | DAXX | SOCS2 | S100A1 | ANKHD1 | PDIA4 | ZNF638 | DLEU2 | LRRC1 | C20orf67 |
| XRCC6 | CLINT1 | PCF11 | BARD1 | ELOVL5 | PFKL | RABL4 | SIRPA | NOTCH1 | SPSB3 |
| HSPA1A | SCAMP3 | PDCD6 | SNX15 | REEP5 | PTBP1 | C21orf21 | KPNB1 | OBFC2B | RAB9A |
| C20orf191 | RNF13 | MET | SNRPG | PRPF6 | WDR37 | MAP3K9 | SOX15 | C1orf66 | ZNF143 |
| NCOR1 | ATXN2L | PTPN18 | HDAC9 | H1F0 | ALAS2 | ABHD5 | IL9R | CCDC59 | LOC388969 |
| RAB11A | VPS26A | GTF2H4 | FXYD2 | DDX18 | FBL | TWF1 | tcag7.350 | TPRKB | MED16 |
| SSRP1 | TIMM17A | ZKSCAN5 | APOM | NXF1 | PLAU | PRKCSH | RPL15 | C2orf33 | KLHL24 |
| TRIM28 | NEDD8 | STS | SDS | SEPHS1 | RNGTT | AKR7A2 | GART | PBK | PDXDC2 |
| UBE2V1 | UQCRC1 | MRPS14 | HSD17B6 | RBM10 | MYO1A | EEF1D | PIP5K1B | MRPL11 | PAPOLA |
| EIF1AX | ALDH3A2 | FANCA | FMO5 | HNRPH3 | EIF4B | FETUB | COX7C | RNF128 | FLII |
| PSMF1 | PPFIA1 | R3HDM2 | ZC3H11A | UCP2 | UBR4 | HIST1H2AM | ITM2B | C3orf14 | SGEF |
| GOLGB1 | COPS3 | SMN1 | KLRK1 | PINK1 | RANBP5 | ZKSCAN1 | C20orf43 | C1orf159 | YES1 |
| SMARCC1 | TJP2 | SMN2 | SLC7A4 | SYNCRIP | COL4A1 | ZNF131 | GSTK1 | GTPBP1 | CNOT2 |
| RBBP7 | NUP153 | NFKBIE | GPA33 | MDH2 | WNK1 | SFRS2B | HN1 | C18orf22 | DDEFL1 |
| ARF4 | IGBP1 | ARG2 | FOXJ3 | SF3B4 | KIAA0182 | VAMP2 | RDH11 | C17orf68 | POLM |
| CSE1L | PYCR1 | GNB5 | SOCS6 | AQP1 | RHOB | YTHDC1 | ENAH | COPZ2 |  |

**Table B:** p-values of correlation between expressions of 120 lapatinib insensitivity markers and response to five drugs that target HER2 and/or EGFR. p-values which are less than 0.05 are highlighted in grey. P-values associated with less that 10% FDR are shown in bold. Genes having correlation at less than 10% FDR with cell response to at-least one drug are marked with asterics. There are two columns corresponding to Afatinib, this is because GDSC dataset {Yang, 2013 #295} has two sets of data corresponding to lapatinib response.

| Gene Symbol | Erlotinib | Lapatinib | EKB-569 | Afatinib | Afatinib | CP724714 |
| --- | --- | --- | --- | --- | --- | --- |
| GPATCH4* | 0.9283 | 0.3443 | 0.1132 | 0.0941 | 0.0025 | 0.0489 |
| SOX15 | 0.5432 | 0.5637 | 0.1841 | 0.0194 | 0.2766 | 0.0696 |
| DLEU2 | 0.2957 | 0.5046 | 0.2114 | 0.0425 | 0.0293 | 0.1196 |
| PKNOX1 | 0.4823 | 0.0468 | 0.6402 | 0.919 | 0.5934 | 0.0633 |
| SDS* | 0.3341 | 0.9597 | 0.3531 | 0.0531 | **0.0103** | 0.2015 |
| GTPBP1* | 0.909 | 0.3687 | 0.4837 | **0.0005** | **0.0009** | **0.0015** |
| SCAMP3 | 0.5224 | 0.9116 | 0.5583 | 0.0105 | 0.0276 | 0.0694 |
| SAR1A | 0.0197 | 0.8083 | 0.9916 | 0.1773 | 0.4606 | 0.7713 |
| LYPD3* | 0.5875 | 0.3441 | 0.2118 | **0.0004** | **0.0016** | 0.0094 |
| MRPL11 | 0.0187 | 0.9663 | 0.8342 | 0.3671 | 0.1742 | 0.6403 |
| IGHA1 | 0.3205 | 0.7238 | 0.2294 | 0.042 | 0.0805 | 0.1718 |
| BARD1 | 0.4631 | 0.3095 | 0.7564 | 0.0454 | 0.0598 | 0.0932 |
| LYN | 0.0971 | 0.0875 | 0.5118 | 0.0919 | 0.017 | 0.0101 |
| PIGQ | 0.2672 | 0.3065 | 0.6537 | 0.0453 | 0.0803 | 0.0058 |
| NCAPG2 | 0.8044 | 0.7497 | 0.2862 | 0.0381 | 0.0627 | 0.1014 |
| CLTB | 0.0428 | 0.1768 | 0.6829 | 0.45 | 0.2549 | 0.2691 |
| MED16 | 0.5482 | 0.0618 | 0.1125 | 0.1333 | 0.1238 | 0.0103 |
| LRRC1 | 0.1316 | 0.0547 | 0.0045 | 0.2064 | 0.0276 | 0.1701 |
| CTLA4* | 0.8904 | 0.7691 | 0.498 | 0.1119 | **0.0088** | 0.0888 |
| LDLRAP1* | 0.3894 | 0.0774 | 0.0374 | **0.0043** | **0.0003** | 0.0056 |
| BAZ1A | 0.27 | 0.8768 | 0.6038 | 0.0438 | 0.0634 | 0.4268 |
| DYNLL1 | 0.9195 | 0.8159 | 0.9403 | 0.0191 | 0.0864 | 0.528 |
| HADHA | 0.0182 | 0.8505 | 0.9824 | 0.1374 | 0.6189 | 0.9704 |
| MMP14 | 0.1316 | 0.4893 | 0.2291 | 0.0538 | 0.0354 | 0.0685 |
| NCOR1 | 0.1967 | 0.1799 | 0.2264 | 0.2325 | 0.0379 | 0.0394 |
| ACRV1* | 0.1525 | 0.0907 | 0.3216 | **0.0002** | **0.0043** | 0.0206 |
| GART | 0.8381 | 0.7731 | 0.3037 | 0.0276 | 0.2145 | 0.1518 |
| CHST3 | 0.0016 | 0.2694 | 0.3442 | 0.5862 | 0.0356 | 0.0745 |
| ASCL1 | 0.98 | 0.3775 | 0.1803 | 0.1199 | 0.3904 | 0.0172 |
| RECQL5* | 0.9265 | 0.0175 | 0.0582 | 0.0154 | **0.0021** | 0.0326 |
| GPA33 | 0.6592 | 0.7741 | 0.0782 | 0.1775 | 0.0959 | 0.0335 |
| TP53TG1 | 0.0361 | 0.7054 | 0.6804 | 0.5703 | 0.1816 | 0.2702 |
| UCP2* | 0.2542 | 0.0376 | 0.0007 | **0.0006** | **0.0001** | **0.0002** |
| SRSF10 | 0.3023 | 0.4743 | 0.5701 | 0.0121 | 0.1241 | 0.2404 |
| FMO5 | 0.6082 | 0.4448 | 0.0627 | 0.0653 | 0.0211 | 0.4166 |
| SNX15 | 0.7468 | 0.4151 | 0.0396 | 0.0238 | 0.2964 | 0.3796 |
| PDCD6 | 0.701 | 0.0289 | 0.3644 | 0.7298 | 0.5246 | 0.6112 |
| UBE2D3 | 0.0087 | 0.5969 | 0.7843 | 0.478 | 0.7296 | 0.8751 |
| IRS2 | 0.8052 | 0.336 | 0.9509 | 0.1642 | 0.0277 | 0.0842 |
| IPO5 | 0.3129 | 0.242 | 0.0388 | 0.7017 | 0.2475 | 0.1407 |
| MAP3K9 | 0.7568 | 0.2708 | 0.3071 | 0.0226 | 0.0762 | 0.1615 |
| S100A1* | **0** | 0.9075 | 0.3914 | 0.0093 | 0.3382 | 0.891 |
| SNRPG | 0.3682 | 0.3446 | 0.5764 | 0.3466 | 0.1502 | 0.0143 |
| SREK1 | 0.1029 | 0.5135 | 0.6138 | 0.0458 | 0.1598 | 0.3259 |
| KPNB1* | 0.6292 | 0.0277 | 0.2432 | **0.0019** | **0.0001** | 0.0128 |
| NEUROD4 | 0.9158 | 0.3835 | 0.967 | 0.0474 | 0.0599 | 0.6131 |
| ANKRD10 | 0.0084 | 0.5351 | 0.3976 | 0.8549 | 0.4037 | 0.5974 |
| SOCS6 | 0.9109 | 0.1353 | 0.1974 | 0.1389 | 0.0121 | 0.0091 |
| FANCA* | 0.0881 | 0.3345 | 0.3052 | 0.1184 | **0.0108** | 0.0531 |
| FOXN2 | 0.8815 | 0.1635 | 0.4844 | 0.3706 | 0.2159 | 0.0395 |
| REEP5* | 0.4081 | 0.1043 | 0.5573 | 0.0198 | **0.0103** | 0.0657 |
| CNOT2 | 0.0046 | 0.9023 | 0.727 | 0.4758 | 0.5669 | 0.4105 |
| BCL3* | 0.4843 | 0.234 | 0.1116 | 0.0163 | **0.0028** | 0.0037 |
| OGT | 0.4436 | 0.1305 | 0.0762 | 0.1007 | 0.0207 | 0.2726 |
| UFD1L | 0.9258 | 0.0385 | 0.1303 | 0.5705 | 0.1076 | 0.0468 |
| ALAS2* | 0.3584 | 0.3772 | 0.187 | 0.0073 | **0.0065** | 0.0135 |
| ITGB5 | 0.9901 | 0.285 | 0.8014 | 0.1541 | 0.0383 | 0.3939 |
| VBP1 | 0.5179 | 0.3221 | 0.2209 | 0.0082 | 0.0249 | 0.0734 |
| PTP4A1 | 0.2909 | 0.1484 | 0.1073 | 0.2958 | 0.1189 | 0.0475 |
| PTP4A2 | 0.0329 | 0.369 | 0.8957 | 0.2523 | 0.6445 | 0.8153 |
| POLR1D* | 0.7574 | 0.0025 | 0.0561 | 0.1217 | **0.0075** | 0.0844 |
| CYB5B | 0.872 | 0.7151 | 0.6658 | 0.0642 | 0.0101 | 0.0388 |
| PFKL | 0.9039 | 0.0345 | 0.6616 | 0.278 | 0.135 | 0.038 |
| EIF3A | 0.6498 | 0.532 | 0.0444 | 0.9317 | 0.9674 | 0.5354 |
| FAM89B | 0.4032 | 0.213 | 0.1387 | 0.2919 | 0.1379 | 0.0276 |
| CPNE1 | 0.285 | 0.5961 | 0.6608 | 0.5009 | 0.0453 | 0.7214 |
| DAG1 | 0.3551 | 0.8868 | 0.7072 | 0.0332 | 0.0511 | 0.626 |
| UBAP2L | 0.0821 | 0.9444 | 0.5023 | 0.0433 | 0.7434 | 0.0575 |
| TNS1* | 0.9483 | 0.2475 | 0.9786 | 0.02 | 0.0336 | **0.0018** |
| CMC2 | 0.455 | 0.2657 | 0.6461 | 0.0386 | 0.0521 | 0.0087 |
| ECH1* | 0.5515 | 0.0184 | 0.2412 | 0.0345 | **0.0113** | 0.1961 |
| ZNF710* | 0.4919 | 0.0555 | 0.1263 | **0.0002** | **0** | **0.0004** |
| SH2D2A | 0.0393 | 0.1508 | 0.4724 | 0.915 | 0.4434 | 0.4365 |
| PIK3R3 | 0.0345 | 0.8196 | 0.9871 | 0.0702 | 0.1292 | 0.4811 |
| MAGEC2 | 0.4935 | 0.0384 | 0.996 | 0.7085 | 0.7668 | 0.7257 |
| SRPK2 | 0.6142 | 0.1936 | 0.0429 | 0.3114 | 0.7806 | 0.5118 |
| SDC1* | 0.0609 | 0.223 | 0.0798 | **0** | **0** | **0.0022** |
| ITM2B | 0.0124 | 0.5077 | 0.9125 | 0.3726 | 0.9681 | 0.9358 |
| GPAA1* | 0.3786 | 0.0532 | 0.2654 | **0.0026** | **0.0038** | 0.0169 |
| HINT1 | 0.4798 | 0.4259 | 0.0226 | 0.1526 | 0.0511 | 0.3715 |
| CCR8* | 0.2675 | 0.0117 | 0.0241 | 0.1398 | **0.0088** | **0.0022** |
| RHOB* | 0.9897 | 0.0198 | 0.147 | 0.0071 | **0.0033** | **0.0009** |
| EXOSC4* | 0.0717 | 0.0497 | 0.1538 | 0.0074 | **0.0085** | 0.0112 |
| RPA1 | 0.6708 | 0.0139 | 0.1862 | 0.0885 | 0.0527 | 0.023 |
| VAX2 | 0.0272 | 0.9937 | 0.0453 | 0.6781 | 0.886 | 0.5013 |
| MPHOSPH9 | 0.5504 | 0.2117 | 0.3399 | 0.0186 | 0.0193 | 0.0347 |
| SGSM3 | 0.9668 | 0.7623 | 0.5826 | 0.0324 | 0.04 | 0.0643 |
| NEDD4 | 0.1981 | 0.7149 | 0.1756 | 0.0098 | 0.1104 | 0.2024 |
| SGSM2 | 0.4458 | 0.8543 | 0.5342 | 0.0178 | 0.0284 | 0.071 |
| TTC3 | 0.0082 | 0.9762 | 0.8441 | 0.3929 | 0.861 | 0.9174 |
| FOXA1* | 0.2772 | 0.1412 | 0.2953 | 0.027 | **0.0026** | 0.0085 |
| GEMIN2 | 0.3459 | 0.1954 | 0.1502 | 0.2369 | 0.0378 | 0.1311 |
| EP300 | 0.5841 | 0.4779 | 0.0074 | 0.2305 | 0.1441 | 0.0157 |
| SIRPA | 0.9731 | 0.5426 | 0.6992 | 0.0205 | 0.302 | 0.6823 |
| PRPF6 | 0.1476 | 0.5256 | 0.6654 | 0.0151 | 0.3665 | 0.0958 |
| SCNN1A* | 0.1015 | 0.5241 | 0.0513 | **0.0028** | **0.0002** | 0.1883 |
| MASP1 | 0.1228 | 0.4447 | 0.8661 | 0.0263 | 0.0369 | 0.017 |
| CUZD1 | 0.2851 | 0.2492 | 0.8494 | 0.0165 | 0.0514 | 0.0538 |
| ASIC1 | 0.0422 | 0.6717 | 0.4889 | 0.2749 | 0.4428 | 0.5476 |
| TFAP2C | 0.2696 | 0.6244 | 0.7646 | 0.0268 | 0.2273 | 0.2365 |
| VDR* | 0.6103 | 0.0322 | 0.0315 | 0.0542 | **0.0041** | **0.0008** |
| SUPT5H | 0.5778 | 0.3954 | 0.0317 | 0.3658 | 0.2934 | 0.1516 |
| GNB5* | 0.8944 | 0.2173 | 0.0554 | **0.0004** | **0.0008** | 0.0064 |
| LSM14B | 0.0268 | 0.9956 | 0.8867 | 0.9726 | 0.9201 | 0.9655 |
| HMGB1 | 0.6753 | 0.0233 | 0.0588 | 0.5123 | 0.1324 | 0.2371 |
| ELAVL1 | 0.0928 | 0.757 | 0.5949 | 0.1143 | 0.0469 | 0.5949 |
| PPP2R5B | 0.2491 | 0.0389 | 0.7592 | 0.6155 | 0.5741 | 0.3475 |
| EEF1D | 0.3473 | 0.0522 | 0.5133 | 0.0221 | 0.0658 | 0.0735 |
| RBM47* | 0.4766 | 0.1901 | 0.0917 | 0.0161 | **0.0093** | 0.0653 |
| EFS | 0.2417 | 0.399 | 0.1174 | 0.4103 | 0.4669 | 0.0306 |
| MAPK1 | 0.7517 | 0.7806 | 0.6818 | 0.044 | 0.1425 | 0.6122 |
| PDLIM4 | 0.5034 | 0.4575 | 0.0733 | 0.0229 | 0.015 | 0.1172 |
| GALNT7* | 0.9052 | 0.0181 | 0.0626 | **0.002** | **0** | 0.0075 |
| POLM* | 0.8368 | 0.2365 | 0.4039 | 0.0077 | 0.013 | **0.0014** |
| ATP2B4 | 0.0737 | 0.2596 | 0.3469 | 0.1811 | 0.0199 | 0.0252 |
| HN1 | 0.2489 | 0.0064 | 0.0867 | 0.3794 | 0.6893 | 0.0328 |
| MLPH* | 0.4314 | 0.1034 | 0.2321 | 0.0324 | **0.0057** | 0.0124 |
| TOMM70A | 0.586 | 0.2399 | 0.8853 | 0.3182 | 0.0694 | 0.0461 |
| TAF5 | 0.6567 | 0.1499 | 0.1256 | 0.1165 | 0.0436 | 0.1295 |
| PAXBP1 | 0.1375 | 0.6027 | 0.4607 | 0.0279 | 0.3467 | 0.5019 |

**Table C:** log-rank p-values of survival data analysis. First column from the left contains gene names, second column contains Affymatrix probset Id for each gene. The third, fourth and fifth columns contain the log-rank p-values, calculated by the kmplot website, which represents the extent of correlation between gene expression and the recurrence free survival of the BC patients.

| Gene Symbol | AffyID | | HER2-ve (800) | HER2+ (160) | Basal |
| --- | --- | --- | --- | --- | --- |
| GPATCH4 -* | 224634_at | | 0.0052 | 0.49 | 0.00012 |
| SDS-+* | | 205695_at | 0.027 | 0.016 | 0.0028 |
| GTPBP1* | 219357_at | | 0.29 | 0.46 | 1.00E-07 |
| LYPD3-+ | 204952_at | | 0.0023 | 0.034 | 0.28 |
| CTLA4* | 236341_at | | 0.11 | 0.073 | 0 |
| LDLRAP1 | 221790_s_at | | 0.27 | 0.25 | 0.15 |
| ACRV1-* | 208013_s_at | | 0.032 | 0.079 | 2.50E-07 |
| RECQL5+* | 221686_s_at | | 0.34 | 0.016 | 3.90E-06 |
| UCP2-+* | 208998_at | | 0.046 | 0.045 | 1.50E-06 |
| S100A1-+ | 205334_at | | 0.0056 | 0.00017 | 0.18 |
| KPNB1-+* | 208975_s_at | | 0.027 | 0.0029 | 0.0053 |
| FANCA-* | 203805_s_at | | 0.0014 | 0.24 | 2.40E-05 |
| REEP5-+* | 208873_s_at | | 0.00012 | 0.037 | 0.00067 |
| BCL3- | 204908_s_at | | 0.026 | 0.31 | 0.26 |
| ALAS2-+* | 211560_s_at | | 0.0011 | 0.018 | 2.10E-09 |
| POLR1D+ | 224874_at | | 0.12 | 3.00E-04 | 0.48 |
| TNS1-+ | 221748_s_at | | 0.038 | 6.10E-06 | 0.33 |
| ECH1-+ | 200789_at | | 0.032 | 1.70E-05 | 0.15 |
| ZNF710-+* | 239700_at | | 0.074 | 0.0043 | 0.0036 |
| SDC1-+* | 201286_at | | 0.0076 | 0.033 | 0.0011 |
| GPAA1-+* | 201618_x_at | | 7.10E-07 | 0.0024 | 0.057 |
| CCR8* | 208059_at | | 0.22 | 0.27 | 1.10E-07 |
| RHOB-+* | 212099_at | | 0.00013 | 0.0038 | 0.014 |
| EXOSC4-+* | 218695_at | | 0.023 | 0.0011 | 0.0053 |
| FOXA1-+* | 204667_at | | 0.00022 | 0.02 | 0.011 |
| SCNN1A-* | 203453_at | | 0.00063 | 0.34 | 0.02 |
| VDR-+* | 204254_s_at | | 0.034 | 0.0035 | 0.0014 |
| GNB5-+ | 204000_at | | 1.40E-05 | 0.0067 | 0.17 |
| RBM47-* | 218035_s_at | | 0.0052 | 0.26 | 0.062 |
| GALNT7-+* | 218313_s_at | | 0.0011 | 0.019 | 0.032 |
| POLM- | 222238_s_at | | 0.0011 | 0.32 | 0.083 |
| MLPH-+* | 218211_s_at | | 3.80E-06 | 0.03 | 0.058 |
|  |  | |  |  |  |


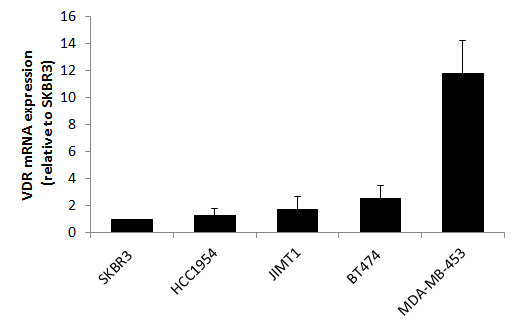


**Figure A.** VDR mRNA expression levels in a panel of HER2 positive breast cancer cell lines relative to SKBR3 cells . qRT-PCR was performed using triplicate technical replicates of 2 biological replicates.

**Figure B.** VDR mRNA expression levels in HCC1954 lapatinib resistant cells (HCC1954-L) relative to HCC1954 parental cells. qRT-PCR was performed using triplicate technical replicates of 2 biological replicates.
